# Supplementary material for: Prehospital major incident management: how do training and real-life situations relate? A qualitative study
Source: BMJ Open. 2021 Sep 23;11(9):e048792. doi: 10.1136/bmjopen-2021-048792 (PMC8461747; doi:10.1136/bmjopen-2021-048792)
Supplement: Author's manuscript [file bmjopen-2021-048792.draft_revisions.pdf]

BMJ Open is committed to open peer review. As part of this commitment we make the peer review history of every article we publish publicly available.

When an article is published we post the peer reviewers' comments and the authors' responses online. We also post the versions of the paper that were used during peer review. These are the versions that the peer review comments apply to.

The versions of the paper that follow are the versions that were submitted during the peer review process. They are not the versions of record or the final published versions. They should not be cited or distributed as the published version of this manuscript.

BMJ Open is an open access journal and the full, final, typeset and author-corrected version of record of the manuscript is available on our site with no access controls, subscription charges or pay-per-view fees (http://bmjopen.bmj.com).

If you have any questions on BMJ Open's open peer review process please email info.bmjopen@bmj.com

BMJ Open

Prehospital major incident management: how does training and authentic situations relate?

Journal:	*BMJ Open*
Manuscript ID	bmjopen-2021-048792
Article Type:	Original research
Date Submitted by the Author:	07-Jan-2021
Complete List of Authors:	Hugelius, Karin; Orebro University, Faculty of Medicine and Health<br>Edelbring, Samuel; Örebro University, Faculty of Medicine and Health<br>Blomberg, Karin; Örebro University, Faculty of Medicine and Health
Keywords:	ACCIDENT & EMERGENCY MEDICINE, TRAUMA MANAGEMENT, HEALTH SERVICES ADMINISTRATION & MANAGEMENT

SCHOLARONE™
Manuscripts

I, the Submitting Author has the right to grant and does grant on behalf of all authors of the Work (as defined in the below author licence), an exclusive licence and/or a non-exclusive licence for contributions from authors who are: i) UK Crown employees; ii) where BMJ has agreed a CC-BY licence shall apply, and/or iii) in accordance with the terms applicable for US Federal Government officers or employees acting as part of their official duties; on a worldwide, perpetual, irrevocable, royalty-free basis to BMJ Publishing Group Ltd ("BMJ") its licensees and where the relevant Journal is co-owned by BMJ to the co-owners of the Journal, to publish the Work in this journal and any other BMJ products and to exploit all rights, as set out in our licence.

The Submitting Author accepts and understands that any supply made under these terms is made by BMJ to the Submitting Author unless you are acting as an employee on behalf of your employer or a postgraduate student of an affiliated institution which is paying any applicable article publishing charge ("APC") for Open Access articles. Where the Submitting Author wishes to make the Work available on an Open Access basis (and intends to pay the relevant APC), the terms of reuse of such Open Access shall be governed by a Creative Commons licence – details of these licences and which Creative Commons licence will apply to this Work are set out in our licence referred to above.

Other than as permitted in any relevant BMJ Author's Self Archiving Policies, I confirm this Work has not been accepted for publication elsewhere, is not being considered for publication elsewhere and does not duplicate material already published. I confirm all authors consent to publication of this Work and authorise the granting of this licence.

For peer review only

Prehospital major incident management: how does training and authentic situations relate?

Karin Hugelius[1], Samuel Edelbring[1], Karin Blomberg[1]

1. Faculty of Medicine and Health, Örebro university, Örebro, Sweden.

Corresponding author:

Karin Hugelius

E-mail: karin.hugelius@oru.se

Address: Örebro university, 70182 Örebro, Sweden

Word count: 4943

For peer review only

For peer review only - http://bmjopen.bmj.com/site/about/guidelines.xhtml

Prehospital major incident management: how does training and authentic situations relate?

ABSTRACT

Objective: The aim of this study was to describe the experiences of prehospital incident commanders acting in a major incident and the relationship between their preparations and authentic events.

Design: An explorative, qualitative design was used.

Setting: Prehospital major incidents in Sweden. Data was collected between December 2019 and August 2020.

Participants: Prehospital incident commanders with experiences from major incident management (n=15).

Methods: Semi structured (n = 27) over-time (one to two interviews/participant within 1 month) were conducted. Data were analysed using inductive thematic analysis.

Results: The conformity between authentic major incidents and preparations was good concerning having sufficient knowledge on some of the operational procedures applied in major incidents. However, the preparations did not allow for the complexity and endurance strategies required in authentic incidents. Neither personal preparations such as mental preparedness nor stress management sufficiently covered in the preparations. To some extent, professional experience (such as training) could compensate for the lack of formal preparations.

Conclusions: To cover for the gaps between preparations and authentic experiences, the preparations in terms of training and exercises should reflect the complexity of a real major incident and stimulate the personal preparations to a large extent. In addition to the development of technical skills and personal preparations, major incident preparations should contribute to building self-knowledge and professional self-confidence to act as a prehospital incident commander.

Keywords: major incidents, prehospital management, disaster preparation, training, ambulance personnel

Strengths and limitations of this study

- The two-session approach used for the interviews stimulated a deeper reflection and rich material to analyse.
- Experiences from many different types of major incidents was gathered, giving a broad perspective on the prehospital incident management.
- The multiprofessional background within the research team added different perspectives to the interpretation of the results.
- The limited number of study participants (n=15) and the Swedish setting can impact the transferability of the results.

For peer review only - http://bmjopen.bmj.com/site/about/guidelines.xhtml

- Some interviews were conducted face to face, and other by phone. This may have influenced the sharing of experiences during the interviews.

BACKGROUND

Major incidents are defined as situations where available resources are insufficient for the immediate need of medical care.[1] Moreover, these challenging situations are often characterized by high levels of uncertainty, dynamic development, and time critical decitionmaking.[2,3] The prehospital management of major incidents is an essential part of the overall medical response that requires specific skills and arrangements, such as an effective incident command.[4] However, structures in on-scene management vary around the world. In Sweden, at least one nurse, often specialized in prehospital emergency care, operates all ambulances. In the case of a major incident or disaster event, the first ambulance personnel arriving at the scene will adopt the positions of prehospital incident commander and medical officer in charge. These two positions may be carried out by either one or two persons. These positions take overall responsibility and have a mandate to lead and organize all aspects of the prehospital medical response, including overall decisions, priorities, and requests, distributing prehospital resources and collaborate with relevant partners on site. In this study, the prehospital incident commander and prehospital medical officer in charge were studied without making any distinction between them, and the term prehospital incident commander[4,5] will be used to cover both positions.

Since major incidents are comparatively rare, many ambulance personnel will not have the possibility of building an extensive processional experience from such events. Therefore, training programmes and exercises are traditionally used to prepare responders[6,7]. Training and exercises provide an opportunity to improve individual and team efforts as well as the decision-making process, test technical equipment and working methods, and increase awareness about the complexity of disaster situations[7,8]. To ensure that ambulance personnel are sufficiently prepared to act as prehospital incident commanders in major incidents, such knowledge is essential. Despite the long history and worldwide use of training and exercises to prepare for real major incidents or disasters, little is actually known about their effects.[6,9] Further, the relationship between preparations and reality is an unexplored area.[10-12]

Therefore, the aim of this study was to explore the relationship between preparations and authentic experiences among prehospital incident commanders´ acting in a major incident.

METHODS

An explorative, qualitative design was used to gain insight into first responders' experiences of major incidents in relation to previous preparations.

Study participants and recruitment

Swedish ambulance personnel who had experience from acting as a prehospital incident manager during a major incident were included in the study. To participate, personnel should have acted as a prehospital incident commander and/or prehospital medical officer during a least one major incident (defined as an incident that had led to a heightened level of response within the health care system) during the previous five years. The study

For peer review only - http://bmjopen.bmj.com/site/about/guidelines.xhtml

participants were recruited from December 2019 until August 2020 by written invitations sent to ambulance services where major incidents had occurred during the last five years. In addition, the same invitation was posted in social media groups and forums aimed specifically at Swedish ambulance personnel. If interested in participating in the study, the participants were asked to access a web page for full written information of the study where they could sign up for the study. Subsequently, the first author (KH) contacted the participants for further information of the study and set a suitable time for the first interviews. All ambulance personnel who volunteered to participate in the study matched the criteria and were thus included.

Data collection procedure

A total of 15 Swedish ambulance personnel were interviewed in 27 individual interviews, conducted in two sessions interviews. The first interview was followed by a second interview within two weeks. This approach was used to ensure that reflections and thoughts that had been raised during the first interview were followed up in the second in order to ensure rich data. Three of the study participants declined a second interview due to personal reasons (such as planned holidays). A semi-structured interview guide was used, based on previous research and developed in discussion with all authors. The questions covered demographic information, previous training and exercises in managing major incidents, and questions regarding the participant's´ experiences from working in such incidents. The interview guide was piloted with two prospective participants, after which only small adjustments were made. Therefore, the pilot interviews were included in the analysis. In addition, all study participants were asked to complete the Swedish version of the Posttraumatic checklist-civilian with a cut-off value of 40 used as an indicator for recommending professional assessment of post-traumatic stress.[13] The first and second session interviews lasted between 24 and 89 min (median 53 min) and 5 and 22 min (median 12 min), respectively. In all, the interview material covered almost 16 hours.

All interviews were conducted by the first author (KH), either by telephone (n=13) or face-to-face (n=2) depending on geographical distances and the Covid 19 pandemic outbreak. Face-to-face interviews were conducted in privacy at the workplace of the participant. Full study information was given, and a written informed consent was obtained from the participants before the first interview. The study was reviewed and approved by the Swedish Ethical Review Authority (ref id 2018:232).

Analysis

The interviews were transcribed in full by a professional transcriber and subsequently checked by the interviewer. The analysis was initially performed by the first author (KH), then reviewed and discussed with the other authors (SE; KB). An inductive approach thematic analysis[14] was used. First, all interviews were read through to gain a sense of the complete data. Features pertaining to the study purpose were then marked, extracted, and coded. Initially, main themes were identified. Within these, themes and subthemes were then formed from the codes, based on similarities and differences between them. Thematic maps[14] were then used to analyse the relations between the main perspectives, themes, and subthemes. Finally, all themes and sub-themes were labelled and described. Finally, a comprehensive summary of the results, showing the relations between the themes, was formulated. Quotations were identified and used to illustrate the findings and increase trustworthiness.

For peer review only - http://bmjopen.bmj.com/site/about/guidelines.xhtml

Patient and public involvement

No patient involved.

RESULTS

Of the 15 ambulance personnel that were interviewed, 10 were male and 5 were female. Their age varied from 29 to 62 years, with a median of 38 years. Their professions were as follows: ambulance nurses with a specialization in ambulance care (n = 7), nurses with a specialization in anaesthesia care (n = 4), and general nurses (n = 4). They had all been deployed within the ambulance services between 1 and 39 years (median of 18 years). All except one was specifically trained in prehospital major incidents management, most commonly in a standardized two-day course consisting of both theoretical and practical training. Their experiences in major incidents covered in the interviews included the following: a bridge collapse, car-, bus-, or train accidents, a vehicle-ramming attack, an aircraft crash, a shooting, an explosion, a chemical incident, and a fire in an elderly home care centre. Some participants had experienced several major incidents. According to the Posttraumatic checklist-civilian, none of the participants exhibited any signs of being traumatized by their experiences.

The following two main themes emerged: *management of authentic events* and *preparations*. Five additional themes were identified: *manage the response, being in charge, operational procedures, personal preparedness*, and *professional experience*. After presenting the themes and subthemes (see Table 1), a summary of the findings and their relations are displayed in Figure 1.

Table 1. Overview of main themes, themes, and subthemes

**Main theme**	**Management of authentic events**		**Preparations**		
**Theme**	Manage the response	Being in charge	Operational procedures	Personal processes	Professional experience
**Sub-themes**	To use a certain methodology	To feel lonely and exposed	Principles and methodology	Mental preparedness	Interpretation of the situation
	To grasp the situation	To be responsible for others	Manage the complexity	Ethical dilemmas	Mediating stress
	To make decisions	To manage stress	Manage endurance	Self-knowledge	Enriched the self-trust
	To improvise	To trust once capability			

Management of authentic major incidents

This main theme described the authentic experiences from acting as a prehospital incident commander in a major incident.

Manage the response

The first information presented to the incident commanders about the major incident was vague, and they had limited information on the extent and character of the situation.

For peer review only - http://bmjopen.bmj.com/site/about/guidelines.xhtml

Therefore, few of them were mentally prepared to deal with a major incident when arriving at the scene.

To gain control of the situation and plan for dynamic development, the incident commanders strived to obtain an overview of the actual event as soon as they arrived at the scene. The importance of stopping and thinking, taking a breath, and assimilating the scene instead of rushing into it was strongly emphasized.

> *"Stop and ... and think. There is always time to stop and think about the whole thing before you get into such a situation. Even if it is chaotic when you arrive, it is worth taking these extra ... to use 20-30 seconds and try to get a picture of the situation and the victims. You should try to make a plan before entering the scene"* (Participant 5)

Several participants referred to a structure and methodology they had been taught to organize the response. Sufficient knowledge on the principles and structure used to manage major incidents were therefore necessarily. Staying with the agreed methodology, especially during the start-up phase, was emphasized.

Sensory impressions (such as visual, hearing, sight, and smell) combined with professional experience and a 'gut feeling' based on knowledge and intuition were used to interpret the situation and plan for consequences and possible developments. In this process, previous experience from both authentic events and training could contribute.

> *"I spontaneously felt in my body that I recognized this as a very difficult, major event and at the same time I was somehow mentally prepared for that something more would happen. So, I started to look for more injured. My intuition told me that there were more [injured] to find".* (Participant 2)

Since the conditions changed every minute, the analysis of consequences and potential development of the incident was a continually ongoing process. Further, the development of the situations was consequently hard to predict, meaning incident commanders had to be constantly prepared to adapt their strategies and actions. Examples of unexpected events could be to find more victims, unexpected changes in the medical conditions, a sudden weather change, or an escalating security situation. The incident commanders strived to be one step ahead and plan for different scenarios, using their professional experience and local knowledge to guide them.

> *"The conditions changed immediately at the triage site when one of the patients had convulsions, from being up walking, he just collapsed and developed general convulsions. I had to change the plan and made the helicopter to take him immediately… "(*Participant 1)

Several decisions had to be taken, which in many cases had to be conducted quickly and only based on fragmentary information. To make wise decisions, the incident commander required good local knowledge (such as geography and logistical aspects). Knowledge on the capacity of nearby hospitals and personal knowledge of the ambulance personnel present at the scene facilitated the management and use of available resources. If local knowledge was lacking, the incident commanders risked making decisions with major consequences, such as wrong or too late decisions concerning the distribution of patients, possible locations for evacuation, and other logistical questions.

Being in charge

For peer review only - http://bmjopen.bmj.com/site/about/guidelines.xhtml

Being an incident commander entailed organising the medical response, leading colleagues, and making decisions that would greatly impact others. The mandate of being an incident commander, which was both formal and informal, implied power and a greater responsibility compared to their normal work. To be trusted and respected, the incident commanders strived to clearly demonstrate their capability to manage the situation. For example, they used calm body language, talked slowly and loudly, and changed their methods of communication to shorter and more concise information compared with in everyday situations. Communication at the scene often relied on face-to-face communication, physical signals such as 'thumbs-up', or eye contact. Sometimes, the incident commander did not fulfil their obligations, according to other colleagues. At such times, colleagues felt frustrated and made them feel obligated to make their own decisions to compensate for the perceived absence of management.

Most often, the decisions were time critical and could have life changing consequences, which caused added tension and ethical dilemmas. The decision making was most often made by the incident commander on their own. Being aware that their decisions and actions could make the difference between life and death made the incident commanders feel lonely and exposed.

> *"We had no one to ask. It was just my colleague and myself. There was no one else. Everything dependent on us. We are used to it, but it's a thing when you have so many people that are depended on you… I think maybe you do not understand this until after quite a few years within this profession. What you as incident commander decide can have huge consequences for others. We make decisions that otherwise the most senior medical doctor inside the hospital make. You need to understand that the responsibility is huge… "(*Participant 12)

Distanced support, such as from a remote senior advisor on commanding an incident, were not always perceived as supportive. If not present at the site, many of the impressions and complexities of the situation were not grasped, because it was difficult for someone not physically present to assimilate all aspects of the situation. Technical means of communication (such as radios or mobile phones) were less supportive than expected, given the chaotic and often noisy environment. Another aspect of remote support was that it could not assist in the very early stages when many of the toughest decisions were being made.

Acting as a manager also implied being responsible for ambulance colleagues. In particular, security decisions such as if (and when) to send colleagues into collapsed buildings, rubble, or hostile environments stressed the incident commanders.

> *"It is a dangerous world …. But if you have chosen to work within the ambulance services, then you need to have courage. Because we are the last outpost in the society in some way. Although it can be scary, and it is dangerous. But we must face it, we are not protected and if you choose this profession then you have to be prepared for that as well. I think it belongs to the profession…"* (Participant 9)

It was emphasized that being an ambulance personnel sometimes implied working in risky environments. However, a general lack of conformity about acceptable risks hampered the decision-making process and sometimes caused conflicts between the incident commander and ambulance personnel. The presence or perceived force in their own stress reactions, and how these reactions limited their cognitive ability surprised the incident commanders.

For peer review only - http://bmjopen.bmj.com/site/about/guidelines.xhtml

For example, the capability to maintain time perception was lost by many of the incident commanders, with the consequence that they would work for many hours without a break. Stress did not only affect the incident commanders, but all personnel present at the site. Sometimes, the incident commanders had to adapt their plans and their leadership style to the reactions exhibited by other ambulance or rescue personnel.

> *"Do we have ambulance personnel on site who are in distress... so that they cannot handle it or... are my colleagues okey? That it also something to consider when you make decisions..."* (Participant 10)

A certain level of self-trust was necessary to enable managing their own feelings and reactions while simultaneously managing the complexity of the situation. If the incident commander felt safe, trusted, and supported by their colleagues, they could focus more on the actual incident management and less on their own reactions and limitations.

Preparations

Within the main theme of preparations, different aspects of preparations and their relation to the authentic experience were described. In addition, one more theme was included with the main theme preparations—professional experience. However, this theme also relates to the themes within the main theme *management of authentic major incidents*. Consequently, it is placed between the two main theme in the graphical representation (Figure 1).

Operational procedures

Within the operational procedures, some aspects were sufficiently covered in the preparations while some were not. The structure and principles, such as how to organize the response, nomenclature, and reporting and triage systems, formed the basis for a structured methodology to manage major incidents.

> *"The moments that I really benefitted from were that you know that there is a structure, and you know who to contact and what to report when"*. (Participant 2)

Being aware of rules and regulations pertaining to the medical response and other stakeholders' mandates was necessary for enhancing good collaboration with those stakeholders engaged. The logistics, including the flow of patients and distribution of victims, were other issues that required specific knowledge. The sessions teaching such frameworks were mainly theoretical, sometimes being enacted in tabletop training. All these formal strategies were sufficiently covered in the training, forming a base for the incident commander on which to rely.

The authentic incidents were found to be more complex and harder to grasp than previous training sessions conveyed. Especially sensory impression and timelines were hard to represent in simulations and thus, training the use of intuition based on such impressions, or authentic stress reactions such as cognitive impairment was difficult to obtain.

Collaboration with other actors at the scene (such as rescue services or the police) was necessary for smooth management at the scene. This collaboration did not only focus on 'what to do' questions such as methodological or strategic, but also on 'how to do it' questions such as ethical considerations or leadership styles. These aspects were seldom discussed as part of the training sessions, causing a gap in the preparations. Most often, the training and exercises focused on the first start-up phase, such as how to act when arriving

For peer review only - http://bmjopen.bmj.com/site/about/guidelines.xhtml

at the scene. However, the authentic major incidents lasted longer time than any training or exercise.

> *"In general, I think, we often talk about the start-up phase, about how we should do when arriving at the scene. But it is just as important to step down and end the mission in a good way. And all in between… to make sure that the right patient comes to the right hospital, that all colleagues are okay, that we have control of the equipment and so, to make sure that the step down is smooth…. We never train that".* (Participant 13)

The importance of train long-term management, stepping down, and perform a closure of the site was emphasised.

Personal processes

While many personal processes were activated during real major incidents, this did not occur during training or exercises. Sensory impressions such as sights, sounds, smells, or the general life and death awareness of actual victims compared to a manikin were some of these. Such impressions increased the perceived stress in a way that was hard to attain in simulations.

> *"…but when you stand there, there are hearing impressions and you see things…and you smell things… and it is a warm body you hold in your hands and you feel your own pulse when you get stressed in a completely different way than during exercises."* (Participant 11)

Being mentally prepared was an essential part of the personal preparations required to act as a major incident commander. Mainly, being mentally prepared meant a personal process of reflection on what it really meant to be an ambulance personnel in general, and the role as incident commander at a major incident in particular. Since the system implied that almost every ambulance personnel on duty could arrive first to a major incident and becoming the incident commander, the mental preparedness needed to be part of the everyday preparations when entering their shift.

> *"You are never prepared when you come to work in the morning to be standing in the middle of a bridge collapse with 10 to 15 injured five hours later. You are not personal prepared for that. But as a professional, I need to be mentally prepared and think it though on beforehand. I have to think " how would I act if this happened". That's your own responsibility, and a good way to preparing yourself…."* (Participant 1)

Being aware of one´s own personal and professional qualities and limitations, and preparing to feel exposed, lonely, and powerless, were also part of the mental preparations. It was also stressed that preparing for such feelings involved listening and learning from others with experience of authentic events, in addition to using one's own imagination.

Self-knowledge including being aware of how others perceived one's leadership and appearance, personal attributes, and discussions on ethical dilemmas faced when managing real incidents were all highlighted as missing parts of the training. The design and implementation of training affected personal preparations in both positively and negatively. By acquiring a perceived feeling of having succeeded, rather than failing and learning from the mistakes, personal growth could be obtained during the training that could be used in authentic events. By providing the chance to repeat one's performance until success was achieved, and by positive coaching and feedback during the training session, positive and increased self-esteem could be promoted. If the opportunity was provided to repeat the

For peer review only - http://bmjopen.bmj.com/site/about/guidelines.xhtml

performance after feedback and in-depth reflections, a final feeling of having succeeded could be obtained. Such cognitive and physical memories could increase the possibility of acting correctly by instinct when faced with a similar challenge again.

Professional experience

The theme professional experiences was part of both the preparations but also served as a mediator for the gap between authentic events and preparations. Since major incidents were unusual events, many years of deployment within the ambulance service was required to gain sufficient experience. Professional experience related to experiences from authentic incidents could to some extent compensate for a lack of formal preparations. Having sufficient professional experience as ambulance personnel in an everyday perspective, specifically from acting as a prehospital medical commander in both minor and major incidents, was of great importance to be able to function in such a position during a major incident.

> *“You need experience, long experience. And you have to be able to manage much more than what they say on the course ... that is not enough* […] *You need to take so much into consideration; geography, roles, laws and things like that. Then you need to be calm and confident in order to get people to act as you would like them to. And ...so that they trust you….”* (Participant 8)

Professional experience added value when analysing potential development of the situation, when making decisions under pressure, and for bearing responsibility commensurate with the role. In addition, professional experience enriched the self-trust and tolerance for one’s own limitations and balanced feelings of being exposed, which made the incident commander feel more comfortable in the situation.

> *“now afterwards I can feel like "I fix this " so I more self-confident in myself […] I actually do the right thing and think the right things in situations like this. It was very positive for me; it has made me feel more confident in my professional role”* (Participant 5).

Also, experiences from major incidents and from working within the ambulance services in general contributed to tolerate and mediate perceived stress. When acting as an incident commander over a longer period of time or repeatedly, professional growth and self-confidence occurred.

A summary of the themes and the relation between the findings are shown in Figure 1.

---Please insert Figure 1 about here—

Figure 1; Overview of the comprehensive results

DISCUSSION

This study identified what areas that needs further attention and applied guidance on how to prepare prehospital major incident commanders more effectively in the future. The conformity between authentic major incidents and preparations was good concerning having sufficient preparedness on operational procedures such as organization, reporting structure and triage methods. However, the preparations did not fully prepare for the

For peer review only - http://bmjopen.bmj.com/site/about/guidelines.xhtml

complexity and endurance strategies required in authentic major incidents. Neither the incident commanders were prepared for personal processes including stress management, ethical dilemmas and self-knowledge required in authentic situations. To some extent, professional experience could compensate for the lack of training. However, incident managers requested further, and to some extent different, preparations to incorporate operational procedures, professional experiences, and personal preparedness to match the complexity and demands of authentic major incidents.

The incident commanders described how they used information from many sources in addition to sensory impressions and their own experience to interpret the situation, predict development, and make decisions. This process corresponds to the concept of situation awareness, as in the perception and understanding of the situation and its meaning[15]. Developing situational awareness is crucial for responding adequately. However, this study demonstrated that this complex process was hard to grasp in training or exercises, since the these were not perceived as complex as reality. In particular, sensory impressions were hard to simulate, even though they have been found essential for assessing a major incident situation[16]. Neither the skill to improvise was sufficiently covered in the preparations. Since improvisation is often used in emergency management[15,17], its value needs to be further highlighted in the preparations.

The structure and method used for major incident management rely on a command and control paradigm aimed at controlling the situation. This type of methodology has long been considered the 'gold standard' for managing such incidents. However, this approach has been criticized for being unrealistic because it is seldom possible to control major incidents to such extent that the models suggest[18]. As found in this study, the assumption of being able to control a major incident in all aspects might cause doubts in one´s capacity and feelings of being exposed and vulnerable if there is a failure to gain control of an authentic incident. Such experiences have been described previous by other medical professionals when faced with disasters[19-21]. Therefore, it would appear important to reflect on how to balance striving to control and accepting uncontrollable dimensions of a major incident in the preparations.

One question that caused a burden to the commanders was the responsibility for safety and security of colleagues. The same issue has been described among prehospital incident commanders during tunnel incidents[22] and terrorist attacks[16]. During the interviews, reflections about acceptable levels of risks, formal and moral obligations, legal and professional responsibilities of ambulance personnel in general were raised, as well as such dilemmas in the specific context of a major incident. It became clear that these discussions were an important part of one's professional identity but also the mental preparedness for managing major incidents.

Managing stress was another challenge raised by the participants. Perceived stress is often a reasonable result of the demands to manage a large amount of uncertainty[23]. However, acting in an unfamiliar position, being witness to a large amount of human suffering, managing safety and security issues and high expectations both from oneself and others were also mentioned as stressors. It has been suggested that ambulance personnel need to develop strong self-resilient strategies to manage the stress inherent in both everyday work and critical incidents[24]. This study has shown that acting as a major incident commander adds several dimensions of stress compared to everyday situations. Stress management and

For peer review only - http://bmjopen.bmj.com/site/about/guidelines.xhtml

resources to stay resilient during and after stressful and potentially traumatic events is not only a personal capacity—it is highly dependent on organizational support[25]. Therefore, stress management strategies should be seen as an essential skill that needs to be addressed in the preparations to enhance a resilient response to major incidents.

Professional experience was found to mediate some of the gaps between preparations and the authentic experiences. Since few incident commanders will have the possibility to gain a rich experience from authentic major incidents, trainings and other kinds of preparations is needed. The evaluation and feedback to participants in major incident trainings are often informal[26] or, as stated by the participants, focused on errors and shortcomings. By focusing on positive post-exercise debriefs[26] and by stimulating self-reflection during trainings[27] professional experience and self-confidence can be better enhanced. In addition, it can be suggested that debriefs or other feedback sessions focus not only on technical procedures and skills, but also on interpersonal and personal competences such as leadership, communication skills, and stress management. As suggested by participants in this study, sharing authentic experiences can also enrich personal preparations.

Several suggestions on how to minimize the gap between preparations and authentic major incidents has been reported in this study. However, how to design and conduct effective training and exercises and what methods should be used to develop technical skills and build the necessary professional and personal preparedness is a challenge that need to be further investigated.

Limitations

As in all qualitative studies, the results of this study rely on a sample of experiences gained from personal experiences. The two-session approach used was found to stimulate a deeper reflection and rich material to analyse. The discussion during the analysis between all three authors provided a broad basis for the analysis, enriched by different professional backgrounds covering among other things disaster medicine, and crises management, health professions education and professional development.

CONCLUSIONS

Current major incident training prepares somewhat for actual major incident management. To cover for existing gaps between preparations and authentic experiences, future training and exercises should reflect the complexity of a real major incident and stimulate the personal preparations to a larger extent. In addition to the development of technical skills and personal preparations, major incident preparations should contribute to building self-knowledge and professional self-confidence to act as a prehospital incident commander.

Funding

This research received no specific grant from any funding agency in the public, commercial or not-for-profit sectors.

DECLARATIONS

Ethics approval and consent to participate

The study was reviewed and approved by the Swedish Ethical Review Authority (ref id 2018:232).

Availability of data and materials

For peer review only - http://bmjopen.bmj.com/site/about/guidelines.xhtml

The data analysed during the current study are not publicly available due to Swedish laws on research ethics and the ethical approval.

Competing interests
The authors declare that they have no competing interests.

Author contributions
All authors participated in the planning and design of the study. KH conducted all interview and analysed the data, during discussions with SE and KB. KH was the major contributor in writing the manuscript, with the assistance of SE and KB. All authors read and approved the final manuscript.

REFERENCES

1. Lennquist Montán K, Hreckovski B, Dobson B, Örtenwall P, Montán C, Khorram-Manesh A, et al. Development and evaluation of a new simulation model for interactive training of the medical response to major incidents and disasters. *Eur J Trauma Emerg Surg* 2014;40(4):429-43.
2. Steigenberger N. Organizing for the Big One: A Review of Case Studies and a Research Agenda for Multi- Agency Disaster Response. *J. Contingencies Crisis Manag*. 2016; 24 (2): 60-72.
3. Hugelius K, Becker J, Adolfsson A. Five Challenges When Managing Mass Casualty or Disaster Situations: A Review Study. *Int J Environ Res Public Health*. 2020;17(9):E3068.
4. Rimstad R, Braut, GS. Literature review on medical incident commander. *Prehosp Disaster Med*. 2015;30(2):205-15.
5. Campion EM, Juillard C, Knudson MM, Dicker R, Cohen MJ, Mackersie R, et al. Reconsidering the Resources Needed for Multiple Casualty Events: Lessons Learned From the Crash of Asiana Airlines Flight 214. *JAMA surgery*. 2016;151(6):512-7.
6. Labrague LJ, Hammad K, Gloe DS, McEnroe-Petitte DM, Fronda DC, Obeidat AA, et al. Disaster preparedness among nurses: a systematic review of literature. *Int Nurs Rev*. 2018;65(01):41-53.
7. Hsu EB, Thomas TL, Bass EB, Whyne D, Kelen GD, Green GB. Healthcare worker competencies for disaster training. *BMC Med Educ*. 2006;6:19.
8. Perry RW, Peterson DM. Disaster Exercise Outcomes for Professional Emergency Personnel and Citizen Volunteers. *J. Contingencies Crisis Manag*. 2004;12(1):64-75.
9. Williams J, Nocera M, Casteel C. The Effectivness of Disaster Training for Health Care Workers: A Systematic Review. *Ann Emerg Med*. 2008;52(3):211-22.
10. Andersson A. In case of emergency. *Collaboration exercises at the bounderis between emergency service organization*. PhD Thesis. University West; Nb 8, Sweden. 2016
11. Gowing JR, Walker KN, Elmer SL, Cummings EA. Disaster Preparedness among Health Professionals and Support Staff: What is Effective? An Integrative Literature Review. *Prehosp Disaster Med*. 2017;32(3):321-8.
12. Legemaate GGA, Burkle F, Bierens JJLM. The Evaluation of Reseach Methods during Disaster Exercises: Applicability for Improving Disatser Health Management. *Prehosp Disaster Med*. 2012;27(1):18-25.

For peer review only - http://bmjopen.bmj.com/site/about/guidelines.xhtml

13. Arnberg F, Michel PO, Johannesson KB. Properties of Swedish Posttraumatic Stress Measures after a Disaster. *J Anxiety Disord*. 2014;28(4):402-9.
14. Braun V, Clarke, V. Using thematic analysis in psychology. *Qual Res Psychol*. 2006;3[2]:77-101.
15. Schakel JK, Wolbers, J. To the edge and beyond: How fast-response organizations adapt in rapidly changing crisis situations. *Hum Relat*. 2019:1-32.
16. Rimstad R, Sollid SJ. A retrospective observational study of medical incident command and decision-making in the 2011 Oslo bombing. *Int J Emerg Med*. 2015;4:8:4.
17. Brandrud AS BM, Brattebø G, Pedersen MJ, Håpnes K, Møller K, Bjorge T, Nyen B, Strauman L, Schreiner A, Haldorsen GS, Bergli M, Nelson E, Morgan TS, Hjortdahl P. Local emergency medical response after a terrorist attack in Norway: a qualitative study. *BMJ Qual Saf*. 2017;26(10):806-816.
18. Boersma F, Ferguson, P., Groeneweegen, J., Wolbers, J. *Beyond the Myth of Control: toward network switching in disaster management*. Proceedings of the 11th International ISCRAM Conference – University Park, May 2014. Pennsylvania, USA; 2014.
19. Hugelius K, Adolfsson A, Ortenwall P, Gifford M. Being both helpers and victims; Health professionals' experiences of working during a natural disaster. *Prehosp Disaster Med*. 2017;32(2):117-23.
20. Sloand E, Ho G, Kub J. Experiences of nurse volunteers in Haiti after the 2010 earthquake. *Res Theory Nurs Pract*. 2013;27(3):193-213.
21. Geisz-Everson MA, Dodd-McCue D, Bennett M. Shared Experiences of CRNAs Who Were on Duty in New Orleans During Hurricane Katrina. *AANA journal*. 2012;80(3):205.12.
22. Hylander J, Saveman BI, Björnstig U, Gyllencreutz L. Prehospital management provided by medical on-scene commanders in tunnel incidents in Oslo, Norway - an interview study. *Scand J Trauma Resusc Emerg Med*. 2019;27(1):78.
23. Peters A, McEwen BS, Friston K. Uncertainty and stress: Why it causes diseases and how it is mastered by the brain. *Prog Neurobiol*. 2017;156:164-88.
24. Clompus SR, Albarran JW. Exploring the nature of resilience in paramedic practice: A psycho-social study. *Int Emerg Nurs*. 2016;28:1-7.
25. Southwick SM, Charney DS. Resilience. *The Science of Mastering Life´s Greatest Challanges*. Second ed. New York: Cambridge University Press; 2018.
26. Skryabina E, Riley P, Reedy G, Amlôt R. A scoping review of evaluation methods for health emergency preparedness exercises. *Am J Disaster Med*. 2018;13(2):107-27.
27. Hermelin J, Bengtsson K, Woltjer R, Trnka J, Thorstensson M, Pettersson J, et al. Operationalising resilience for disaster medicine practitioners: capability development through training, simulation and reflection. *Cogn Technol Work*. 2020;22(3):667-83.

For peer review only - http://bmjopen.bmj.com/site/about/guidelines.xhtml

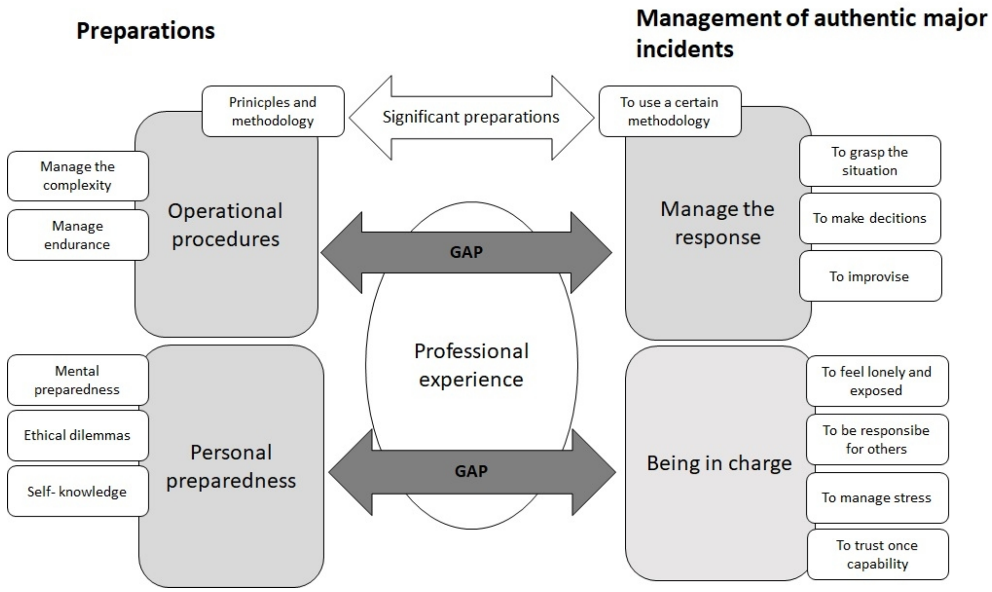

Figure 1; Overview of the results

271x161mm (96 x 96 DPI)

Standards for Reporting Qualitative Research (SRQR)*

http://www.equator-network.org/reporting-guidelines/srqr/

Title and abstract

	Page/line no(s).
**Title** - Concise description of the nature and topic of the study Identifying the study as qualitative or indicating the approach (e.g., ethnography, grounded theory) or data collection methods (e.g., interview, focus group) is recommended	1
**Abstract** - Summary of key elements of the study using the abstract format of the intended publication; typically includes background, purpose, methods, results, and conclusions	1

Introduction

**Problem formulation** - Description and significance of the problem/phenomenon studied; review of relevant theory and empirical work; problem statement	2
**Purpose or research question** - Purpose of the study and specific objectives or questions	2

Methods

**Qualitative approach and research paradigm** - Qualitative approach (e.g., ethnography, grounded theory, case study, phenomenology, narrative research) and guiding theory if appropriate; identifying the research paradigm (e.g., postpositivist, constructivist/ interpretivist) is also recommended; rationale**	2
**Researcher characteristics and reflexivity** - Researchers' characteristics that may influence the research, including personal attributes, qualifications/experience, relationship with participants, assumptions, and/or presuppositions; potential or actual interaction between researchers' characteristics and the research questions, approach, methods, results, and/or transferability	3+11
**Context** - Setting/site and salient contextual factors; rationale**	2
**Sampling strategy** - How and why research participants, documents, or events were selected; criteria for deciding when no further sampling was necessary (e.g., sampling saturation); rationale**	2,3
**Ethical issues pertaining to human subjects** - Documentation of approval by an appropriate ethics review board and participant consent, or explanation for lack thereof; other confidentiality and data security issues	3
**Data collection methods** - Types of data collected; details of data collection procedures including (as appropriate) start and stop dates of data collection and analysis, iterative process, triangulation of sources/methods, and modification of procedures in response to evolving study findings; rationale**	3

For peer review only

For peer review only - http://bmjopen.bmj.com/site/about/guidelines.xhtml

**Data collection instruments and technologies** - Description of instruments (e.g., interview guides, questionnaires) and devices (e.g., audio recorders) used for data collection; if/how the instrument(s) changed over the course of the study	3
**Units of study** - Number and relevant characteristics of participants, documents, or events included in the study; level of participation (could be reported in results)	3,4
**Data processing** - Methods for processing data prior to and during analysis, including transcription, data entry, data management and security, verification of data integrity, data coding, and anonymization/de-identification of excerpts	3
**Data analysis** - Process by which inferences, themes, etc., were identified and developed, including the researchers involved in data analysis; usually references a specific paradigm or approach; rationale**	3
**Techniques to enhance trustworthiness** - Techniques to enhance trustworthiness and credibility of data analysis (e.g., member checking, audit trail, triangulation); rationale**	3

Results/findings

**Synthesis and interpretation** - Main findings (e.g., interpretations, inferences, and themes); might include development of a theory or model, or integration with prior research or theory	4-9
**Links to empirical data** - Evidence (e.g., quotes, field notes, text excerpts, photographs) to substantiate analytic findings	4-9

Discussion

**Integration with prior work, implications, transferability, and contribution(s) to the field -** Short summary of main findings; explanation of how findings and conclusions connect to, support, elaborate on, or challenge conclusions of earlier scholarship; discussion of scope of application/generalizability; identification of unique contribution(s) to scholarship in a discipline or field	9-10
**Limitations** - Trustworthiness and limitations of findings	11

Other

**Conflicts of interest** - Potential sources of influence or perceived influence on study conduct and conclusions; how these were managed	11
**Funding** - Sources of funding and other support; role of funders in data collection, interpretation, and reporting	11

*The authors created the SRQR by searching the literature to identify guidelines, reporting standards, and critical appraisal criteria for qualitative research; reviewing the reference lists of retrieved sources; and contacting experts to gain feedback. The SRQR aims to improve the transparency of all aspects of qualitative research by providing clear standards for reporting qualitative research.

For peer review only

For peer review only - http://bmjopen.bmj.com/site/about/guidelines.xhtml

**The rationale should briefly discuss the justification for choosing that theory, approach, method, or technique rather than other options available, the assumptions and limitations implicit in those choices, and how those choices influence study conclusions and transferability. As appropriate, the rationale for several items might be discussed together.

Reference:

O'Brien BC, Harris IB, Beckman TJ, Reed DA, Cook DA. **Standards for reporting qualitative research: a synthesis of recommendations.** *Academic Medicine*, Vol. 89, No. 9 / Sept 2014 DOI: 10.1097/ACM.0000000000000388

For peer review only

For peer review only - http://bmjopen.bmj.com/site/about/guidelines.xhtml

BMJ Open

Prehospital major incident management: how does training and authentic situations relate? A qualitative study.

Journal:	*BMJ Open*
Manuscript ID	bmjopen-2021-048792.R1
Article Type:	Original research
Date Submitted by the Author:	10-Jun-2021
Complete List of Authors:	Hugelius, Karin; Orebro University, Faculty of Medicine and Health<br>Edelbring, Samuel; Örebro University, Faculty of Medicine and Health<br>Blomberg, Karin; Örebro University, Faculty of Medicine and Health
**Primary Subject Heading**:	Emergency medicine
Secondary Subject Heading:	Emergency medicine
Keywords:	ACCIDENT & EMERGENCY MEDICINE, TRAUMA MANAGEMENT, HEALTH SERVICES ADMINISTRATION & MANAGEMENT

SCHOLARONE™
Manuscripts

I, the Submitting Author has the right to grant and does grant on behalf of all authors of the Work (as defined in the below author licence), an exclusive licence and/or a non-exclusive licence for contributions from authors who are: i) UK Crown employees; ii) where BMJ has agreed a CC-BY licence shall apply, and/or iii) in accordance with the terms applicable for US Federal Government officers or employees acting as part of their official duties; on a worldwide, perpetual, irrevocable, royalty-free basis to BMJ Publishing Group Ltd ("BMJ") its licensees and where the relevant Journal is co-owned by BMJ to the co-owners of the Journal, to publish the Work in this journal and any other BMJ products and to exploit all rights, as set out in our licence.

The Submitting Author accepts and understands that any supply made under these terms is made by BMJ to the Submitting Author unless you are acting as an employee on behalf of your employer or a postgraduate student of an affiliated institution which is paying any applicable article publishing charge ("APC") for Open Access articles. Where the Submitting Author wishes to make the Work available on an Open Access basis (and intends to pay the relevant APC), the terms of reuse of such Open Access shall be governed by a Creative Commons licence – details of these licences and which Creative Commons licence will apply to this Work are set out in our licence referred to above.

Other than as permitted in any relevant BMJ Author's Self Archiving Policies, I confirm this Work has not been accepted for publication elsewhere, is not being considered for publication elsewhere and does not duplicate material already published. I confirm all authors consent to publication of this Work and authorise the granting of this licence.

For peer review only

Prehospital major incident management: how does training and authentic situations relate?

A qualitative study.

Karin Hugelius[1], Samuel Edelbring[1], Karin Blomberg[1]

1. Faculty of Medicine and Health, Örebro university, Örebro, Sweden.

Corresponding author:

Karin Hugelius

E-mail: karin.hugelius@oru.se

Address: Örebro university, 70182 Örebro, Sweden

Word count: 5257

For peer review only

For peer review only - http://bmjopen.bmj.com/site/about/guidelines.xhtml

Prehospital major incident management: how does training and authentic situations relate? A qualitative study.

ABSTRACT

Objective: The aim of this study was to explore the relationship between preparations and authentic experiences among prehospital incident commanders´ acting in a major incident.

Design: An explorative, qualitative design was used.

Setting: Prehospital major incidents in Sweden. Data was collected between December 2019 and August 2020.

Participants: Prehospital incident commanders with experiences from major incident management (n=15). Their authentic experiences covered different major events such as fires, bus accidents, a bridge collapse and terrorist attacks. All but one had participated in a two-day long training focusing on the prehospital management of major incidents. In addition, about half of the participants had participated in simulation exercises, academic courses or other training in the management of major incidents.

Methods: Semi structured (n = 27) over-time (one to two interviews/participant within two weeks) were conducted. Data were analysed using inductive thematic analysis.

Results: The conformity between authentic major incidents and preparations was good concerning having sufficient knowledge on some of the operational procedures applied in major incidents. However, the preparations did not allow for the complexity and endurance strategies required in authentic incidents. Neither personal preparations such as mental preparedness nor stress management sufficiently covered in the preparations. To some extent, professional experience (such as training) could compensate for the lack of formal preparations.

Conclusions: To cover for the gaps between preparations and authentic experiences, the preparations in terms of educational sessions and training exercises should reflect the complexity of a real major incident and stimulate the personal preparations to a large extent. In addition to the development of technical skills and personal preparations, major incident preparations should contribute to building self-knowledge and professional self-confidence to act as a prehospital incident commander.

Keywords: major incidents, prehospital management, disaster preparation, training, ambulance personnel

Strengths and limitations of this study

- The two-session approach used for the interviews stimulated a deeper reflection and rich material to analyse.
- Experiences from many different types of major incidents was gathered, giving a broad perspective on the prehospital incident management.

For peer review only - http://bmjopen.bmj.com/site/about/guidelines.xhtml

- The multiprofessional background within the research team added different perspectives to the interpretation of the results.
- The limited number of study participants (n=15) and the Swedish setting can impact the transferability of the results.
- Some interviews were conducted face to face, and other by phone. This may have influenced the sharing of experiences during the interviews.

BACKGROUND

Major incidents are defined as situations where available resources are insufficient for the immediate need of medical care.[1] Moreover, these challenging situations are often characterized by high levels of uncertainty, dynamic development, and time critical decitionmaking.[2,3] The prehospital management of major incidents is an essential part of the overall medical response that requires specific skills and arrangements, such as an effective incident command.[4] However, structures in on-scene management vary around the world. In Sweden, at least one nurse, often specialized in prehospital emergency care, is on duty in all emergency ambulances. In the case of a major incident or disaster event, the first ambulance personnel arriving at the scene will adopt the positions of prehospital incident commander and medical officer in charge. These two positions may be carried out by either one or two persons. These positions take overall responsibility and have a mandate to lead and organize all aspects of the prehospital medical response, including overall decisions, priorities, and requests, distributing prehospital resources and collaborate with relevant partners on site. In this study, the prehospital incident commander and prehospital medical officer in charge were studied without making any distinction between them, and the term prehospital incident commander[4,5] will be used to cover both positions.

Since major incidents are comparatively rare, many ambulance personnel will not have the possibility of building an extensive processional experience from such events. Therefore, training programmes and exercises are traditionally used to prepare responders[6,7]. Training exercises provide an opportunity to improve individual and team efforts as well as the decision-making process, test technical equipment and working methods, and increase awareness about the complexity of disaster situations[7,8]. To ensure that ambulance personnel are sufficiently prepared to act as prehospital incident commanders in major incidents, such knowledge is essential. Despite the long history and worldwide use of educational sessions and exercises to prepare for real major incidents or disasters, little is actually known about their effects.[6,9] Further, the relationship between preparations and reality is an unexplored area.[10-12]

Therefore, the aim of this study was to explore the relationship between preparations and authentic experiences among prehospital incident commanders´ acting in a major incident.

METHODS

An explorative, qualitative design was used to gain insight into ambulance personnel's experiences of major incidents in relation to previous preparations.

Study participants and recruitment

For peer review only - http://bmjopen.bmj.com/site/about/guidelines.xhtml

Swedish ambulance personnel who had experience from acting as a prehospital incident manager during a major incident were included in the study. To participate, personnel should have acted as a prehospital incident commander and/or prehospital medical officer during a least one major incident (defined as an incident that had led to a heightened level of response within the health care system) during the previous five years. The study participants were recruited from December 2019 until August 2020 by written invitations sent to ambulance services where major incidents had occurred during the last five years. In addition, the same invitation was posted in social media groups and forums aimed specifically at Swedish ambulance personnel. If interested in participating in the study, the participants were asked to access a web page for full written information of the study where they could sign up for the study. Subsequently, the first author (KH) contacted the participants for further information of the study and set a suitable time for the first interviews. All ambulance personnel who volunteered to participate in the study matched the criteria and were thus included.

Data collection procedure

A total of 15 Swedish ambulance personnel were interviewed in 27 individual interviews, conducted in two sessions interviews[13]. The first interview was followed by a second interview within two weeks. This approach was used to ensure that reflections and thoughts that had been raised during the first interview were followed up in the second to ensure rich data[13]. Three of the study participants declined a second interview due to personal reasons (such as planned holidays). A semi-structured interview guide was used, based on previous research and developed in discussion with all authors. The questions used in the first interview covered demographic information, previous educational sessions and training exercises in managing major incidents, and questions regarding the participants´ experiences from working in such incidents. In the second interview, the first interview was followed up on, and the study participants was asked if he or she had any additional thoughts or experiences that had not been mentioned in the first interview. The interview guide was piloted with two prospective participants, after which only small adjustments were made. Therefore, the pilot interviews were included in the analysis. In addition, all study participants were asked to complete the Swedish version of the Posttraumatic checklist-civilian with a cut-off value of 40 used as an indicator for recommending professional assessment of post-traumatic stress.[14] The first and second session interviews lasted between 24 and 89 min (median 53 min) and 5 and 22 min (median 12 min), respectively. In all, the interview material covered almost 16 hours.

All interviews were conducted by the first author (KH), either by telephone (n=13) or face-to-face (n=2) depending on geographical distances and the Covid 19 pandemic outbreak. Face-to-face interviews were conducted in privacy at the workplace of the participant. Full study information was given, and a written informed consent was obtained from the participants before the first interview. The study was reviewed and approved by the Swedish Ethical Review Authority (ref id 2018:232).

Analysis

The interviews were transcribed in full by a professional transcriber and subsequently checked by the interviewer. The analysis was initially performed by the first author (KH), then reviewed and discussed with the other authors (SE; KB). An inductive approach thematic analysis[14] was used and the approach was to identify skills and experiences

For peer review only - http://bmjopen.bmj.com/site/about/guidelines.xhtml

requested and used in authentic incidents, and to relate the preparations to those. First, all interviews were read through to gain a sense of the complete data. Features pertaining to the study purpose were then marked, extracted, and coded. Initially, main themes were identified. Within these, themes and subthemes were then formed from the codes, based on similarities and differences between them. Thematic maps[15] were then used to analyse the relations between the main perspectives, themes, and subthemes. Finally, all themes and sub-themes were labelled and described. Finally, a comprehensive summary of the results, showing the relations between the themes, was formulated. Quotations were identified and used to illustrate the findings and increase trustworthiness.

Patient and public involvement

No patients were involved.

RESULTS

Of the 15 ambulance personnel that were interviewed, 10 were male and 5 were female. Their age varied from 29 to 62 years, with a median of 38 years. Their professions were as follows: ambulance nurses with a specialization in ambulance care (n = 7), nurses with a specialization in anaesthesia care (n = 4), and general nurses (n = 4). They had all been deployed within the ambulance services between 1 and 39 years (median of 18 years). All except one was specifically trained in prehospital major incidents management, most commonly in a standardized two-day course consisting of both theoretical and practical training. Other preparations varied, from no other trainings (except for the above mentioned two days training) (n=8), disaster simulations exercise covering up to 4 hours (n=5), major incident trainings as part of being a voluntary staff within a Non-Governmental Organization (n=2), academic course on disaster medicine (n=1) or major incident exercises within the military services (n=1). Two participants had participated in more than 4 trainings or exercise. None of the participants had participated in simulations covering more than four hours, neither in digital or other kinds of simulation-based activities.

Their experiences in major incidents covered in the interviews included the following: a bridge collapse, car-, bus-, or train accidents, a vehicle-ramming attack, an aircraft crash, a shooting, an explosion, a chemical incident, and a fire in an elderly home care centre. Some participants had experienced several major incidents. According to the Posttraumatic checklist-civilian, none of the participants exhibited any signs of being traumatized by their experiences.

The following three main themes emerged: *skills and experiences needed to manage major incidents, skills significantly covered in preparations* and *gaps in preparations in relation to the demands of authentic major incidents*. Six additional themes were identified: *manage the response, being in charge, frames for management, operational procedures, personal preparedness*, and *professional experience (See Table 1).*

Table 1. Overview of main themes, themes, and subthemes

**Main themes**	**Skills and experiences needed to manage major incidents**	**Skills significantly covered in preparations**	**Gaps in preparations in relation to the demands of major incidents**

For peer review only - http://bmjopen.bmj.com/site/about/guidelines.xhtml

Themes	Manage the response	Being in charge	Frames for management	Operational procedures	Personal processes	Lack of professional experience
Sub-themes	To use a certain methodology	To feel lonely and exposed	Principles and methodology	Manage the complexity	Mental preparedness	Interpretation of the situation
	To grasp the situation	To be responsible for others		Manage endurance	Ethical dilemmas	Mediating stress
	To make decisions	To manage stress			Self-knowledge	
	To improvise	To trust one`s capability				

Skills and experiences needed to manage major incidents

This main theme described the authentic experiences from acting as a prehospital incident commander in a major incident, and skills needed for such management.

Manage the response

The first information presented from the dispatch centre to the incident commanders about the major incident was vague, and they had limited information on the extent and character of the situation. Therefore, few of them were mentally prepared to deal with a major incident when arriving at the scene.

To gain control of the situation and plan for dynamic development, the incident commanders strived to obtain an overview of the actual event as soon as they arrived at the scene. The importance of stopping and thinking, taking a breath, and assimilating the scene instead of rushing into it was strongly emphasized.

> *"Stop and ... and think. There is always time to stop and think about the whole thing before you get into such a situation. Even if it is chaotic when you arrive, it is worth taking these extra ... to use 20-30 seconds and try to get a picture of the situation and the victims. You should try to make a plan before entering the scene"* (Participant 5)

Several participants referred to a structure and methodology they had been taught to organize the response. Sufficient knowledge on the principles and structure used to manage major incidents were therefore necessarily. Examples of such principles were mass casualty triage or reporting algorithms. Staying with the agreed methodology, especially during the start-up phase, was emphasized. However, details in methods and concepts differed from region to region why local knowledge on for example when to report what, and to who was needed.

Sensory impressions (such as visual, hearing, sight, and smell) combined with professional experience and a 'gut feeling' based on knowledge and intuition were used to interpret the situation and plan for consequences and possible developments. In this process, previous experience from both authentic major incidents and training could contribute.

For peer review only - http://bmjopen.bmj.com/site/about/guidelines.xhtml

> *"I spontaneously felt in my body that I recognized this as a very difficult, major incident and at the same time I was somehow mentally prepared for that something more would happen. So, I started to look for more injured. My intuition told me that there were more [injured] to find".* (Participant 2)

Since the conditions changed every minute, the analysis of consequences and potential development of the incident was a continually ongoing process. Further, the development of the situations was consequently hard to predict, meaning incident commanders had to be constantly prepared to adapt their strategies and actions. Examples of unexpected incidents could be to find more victims, unexpected changes in the medical conditions, a sudden weather change, or an escalating security situation. The incident commanders strived to be one step ahead and plan for different scenarios, using their professional experience and local knowledge to guide them.

> *"The conditions changed immediately at the triage site when one of the patients had convulsions, from being up walking, he just collapsed and developed general convulsions. I had to change the plan and made the helicopter to take him immediately… "(*Participant 1)

Several decisions had to be taken, which in many cases had to be conducted quickly and only based on fragmentary information. To make wise decisions, the incident commander required good local knowledge (such as geography and logistical aspects). Knowledge on the capacity of nearby hospitals and personal knowledge of the ambulance personnel present at the scene facilitated the management and use of available resources. If local knowledge was lacking, the incident commanders risked making decisions with major consequences, such as wrong or too late decisions concerning the distribution of patients, possible locations for evacuation, and other logistical questions.

Being in charge

Being an incident commander entailed organising the medical response, leading colleagues, and making decisions that would greatly impact others. The mandate of being an incident commander, which was both formal and informal, implied power and a greater responsibility compared to their normal work. To be trusted and respected, the incident commanders strived to clearly demonstrate their capability to manage the situation. For example, they used calm body language, talked slowly and loudly, and changed their methods of communication to shorter and more concise information compared with in everyday situations. Communication at the scene often relied on face-to-face communication, physical signals such as 'thumbs-up', or eye contact. Sometimes, the incident commander did not fulfil their obligations, according to other colleagues. At such times, colleagues felt frustrated and made them feel obligated to make their own decisions to compensate for the perceived absence of management.

Most often, the decisions were time critical and could have life changing consequences, which caused added tension and ethical dilemmas. The decision- making was most often made by the incident commander on their own. Being aware that their decisions and actions could make the difference between life and death made the incident commanders feel lonely and exposed.

> *"We had no one to ask. It was just my colleague and myself. There was no one else. Everything dependent on us. We are used to it, but it's a thing when you have so many*

For peer review only - http://bmjopen.bmj.com/site/about/guidelines.xhtml

> *people that are depended on you… I think maybe you do not understand this until after quite a few years within this profession. What you as incident commander decide can have huge consequences for others. We make decisions that otherwise the most senior medical doctor inside the hospital make. You need to understand that the responsibility is huge… "* (Participant 12)

Distanced support, such as from a remote senior advisor on commanding an incident, were not always perceived as supportive. If not present at the site, many of the impressions and complexities of the situation were not grasped, because it was difficult for someone not physically present to assimilate all aspects of the situation. Technical means of communication (such as radios or mobile phones) were less supportive than expected, given the chaotic and often noisy environment. Another aspect of remote support was that it could not assist in the very early stages when many of the toughest decisions were being made.

Acting as a manager also implied being responsible for ambulance colleagues. In particular, security decisions such as if (and when) to send colleagues into collapsed buildings, rubble, or hostile environments stressed the incident commanders.

> *"It is a dangerous world …. But if you have chosen to work within the ambulance services, then you need to have courage. Because we are the last outpost in the society in some way. Although it can be scary, and it is dangerous. But we must face it, we are not protected and if you choose this profession then you have to be prepared for that as well. I think it belongs to the profession…"* (Participant 9)

It was emphasized that being an ambulance personnel sometimes implied working in risky environments. However, a general lack of conformity about acceptable risks hampered the decision-making process and sometimes caused conflicts between the incident commander and ambulance personnel. The presence or perceived force in their own stress reactions, and how these reactions limited their cognitive ability surprised the incident commanders. For example, the capability to maintain time perception was lost by many of the incident commanders, with the consequence that they would work for many hours without a break. Stress did not only affect the incident commanders, but all personnel present at the site. Sometimes, the incident commanders had to adapt their plans and their leadership style to the reactions exhibited by other ambulance or rescue personnel.

> *"Do we have ambulance personnel on site who are in distress… so that they cannot handle it or… are my colleagues okey? That it also something to consider when you make decisions…"* (Participant 10)

A certain level of self-trust was necessary to enable managing their own feelings and reactions while simultaneously managing the complexity of the situation. If the incident commander felt safe, trusted, and supported by their colleagues, they could focus more on the actual incident management and less on their own reactions and limitations.

Skills significantly covered in preparations

Some aspects were sufficiently covered in the preparations. The structure and principles, such as how to organize the response, nomenclature, and reporting and triage systems, formed the basis for a structured methodology to manage major incidents.

> *"The moments that I really benefitted from were that you know that there is a structure, and you know who to contact and what to report when"*. (Participant 2)

For peer review only - http://bmjopen.bmj.com/site/about/guidelines.xhtml

Being aware of rules and regulations pertaining to the medical response and other stakeholders' mandates was necessary for enhancing good collaboration with those stakeholders engaged. The logistics, including the flow of patients and distribution of victims, were other issues that required specific knowledge. The sessions teaching such frameworks were mainly theoretical, sometimes being enacted in tabletop training. All these formal strategies were sufficiently covered in the training, forming a base for the incident commander on which to rely.

Gaps in preparations in relation to the demands of authentic major incidents

Within the theme, gaps in preparations, with regard to the demands of managing an authentic event, was described. The gaps found was related to operational procedures, personal processes and professional experiences.

Operational procedures

The authentic incidents were found to be more complex and harder to grasp than previous training sessions conveyed. Especially sensory impression and timelines were hard to represent in simulations and thus, training the use of intuition based on such impressions, or authentic stress reactions such as cognitive impairment was difficult to obtain.

Collaboration with other actors at the scene (such as rescue services or the police) was necessary for smooth management at the scene. This collaboration did not only focus on 'what to do' questions such as methodological or strategic, but also on 'how to do it' questions such as ethical considerations or leadership styles. These aspects were seldom discussed as part of the training sessions, causing a gap in the preparations. Most often, the training exercises focused on the first start-up phase, such as how to act when arriving at the scene. However, the authentic major incidents lasted longer time than any training or exercise.

> *"In general, I think, we often talk about the start-up phase, about how we should do when arriving at the scene. But it is just as important to step down and end the mission in a good way. And all in between… to make sure that the right patient comes to the right hospital, that all colleagues are okay, that we have control of the equipment and so, to make sure that the step down is smooth.... We never train that".* (Participant 13)

The importance of preparing for train long-term management, stepping down, and perform a closure of the site was emphasised.

Personal processes

While many processes on a personal level were activated during real major incidents, this did not occur during training or exercises. Sensory impressions such as sights, sounds, smells, or the general life and death awareness of actual victims compared to a manikin were some of these. Such impressions increased the perceived stress in a way that was hard to attain in simulations.

> *"…but when you stand there, there are hearing impressions and you see things…and you smell things… and it is a warm body you hold in your hands and you feel your own pulse when you get stressed in a completely different way than during exercises."* (Participant 11)

For peer review only - http://bmjopen.bmj.com/site/about/guidelines.xhtml

Being mentally prepared was an essential part of the personal preparations required to act as a major incident commander. Mainly, being mentally prepared meant a personal process of reflection on what it really meant to be an ambulance personnel in general, and the role as incident commander at a major incident in particular. Since the system implied that almost every ambulance personnel on duty could arrive first to a major incident and becoming the incident commander, the mental preparedness needed to be part of the everyday preparations when entering their shift.

> *"You are never prepared when you come to work in the morning to be standing in the middle of a bridge collapse with 10 to 15 injured five hours later. You are not personal prepared for that. But as a professional, I need to be mentally prepared and think it though on beforehand. I have to think " how would I act if this happened". That's your own responsibility, and a good way to preparing yourself…."* (Participant 1)

Being aware of one´s own personal and professional qualities and limitations, and preparing to feel exposed, lonely, and powerless, were also part of the mental preparations. It was also stressed that preparing for such feelings involved listening and learning from others with experience of authentic major incidents, in addition to using one's own imagination.

Self-knowledge including being aware of how others perceived one's leadership and appearance, personal attributes, and discussions on ethical dilemmas faced when managing real incidents were all highlighted as missing parts of the training. The design and implementation of training affected personal preparations in both positively and negatively. By acquiring a perceived feeling of having succeeded, rather than failing and learning from the mistakes, personal growth could be obtained during the training that could be used in authentic major incidents. By providing the chance to repeat one's performance until success was achieved, and by positive coaching and feedback during the training session, positive and increased self-esteem could be promoted. If the opportunity was provided to repeat the performance after feedback and in-depth reflections, a final feeling of having succeeded could be obtained. Such cognitive and physical memories could increase the possibility of acting correctly by instinct when faced with a similar challenge again.

Lack of professional experience

Since major incidents were unusual events, many years of deployment within the ambulance service was required to gain sufficient experience to manage a major incident. In many situations, the personnel in managing positions at major incidents could be quite new and inexperienced. Professional experience from authentic incidents could, to some extent, compensate for a lack of formal training and preparations. Having sufficient professional experience as ambulance personnel in an everyday perspective, specifically from acting as a prehospital medical commander in both minor and major incidents, was of great importance to be able to function in such a position during a major incident.

> *"You need experience, long experience. And you have to be able to manage much more than what they say on the course ... that is not enough* […] *You need to take so much into consideration; geography, roles, laws and things like that. Then you need to be calm and confident in order to get people to act as you would like them to. And ...so that they trust you…."* (Participant 8)

For peer review only - http://bmjopen.bmj.com/site/about/guidelines.xhtml

Professional experience added value when analysing potential development of the situation, when making decisions under pressure, and for bearing responsibility commensurate with the role.

> *"now afterwards I can feel like "I fix this " so I feel more self-confident […] I actually do the right thing and think the right things in situations like this. It was very positive for me; it has made me feel more confident in my professional role"* (Participant 5).

In addition, professional experience enriched the self-trust and tolerance for one's own limitations and balanced feelings of being exposed, which made the incident commander feel more comfortable in the situation. Lacking professional experiences had the opposite effects. Also, lack of experiences from major incidents and from working within the ambulance services in general reduced the necessary ability of a commander in a major incident to tolerate and manage stress.

DISCUSSION

This study identified what areas that needs further attention and applied guidance on how to prepare prehospital major incident commanders more effectively in the future. The conformity between authentic major incidents and preparations was good concerning having sufficient preparedness on operational procedures such as organization, reporting structure and triage methods. However, the preparations did not prepare for the complexity and endurance strategies required in authentic major incidents. Neither the incident commanders were prepared for personal processes including stress management, ethical dilemmas and self-knowledge required in authentic situations. To some extent, professional experience could compensate for the lack of training. However, incident managers requested further, and to some extent different, preparations to incorporate operational procedures, professional experiences, and personal preparedness to match the complexity and demands of authentic major incidents.

The incident commanders described how they used information from many sources in addition to sensory impressions and their own experience to interpret the situation, predict development, and make decisions. This process corresponds to the concept of situation awareness, as in the perception and understanding of the situation and its meaning[16]. Developing situational awareness is crucial for responding adequately. However, this study demonstrated that this complex process was hard to grasp in training or exercises, since the these were not perceived as complex as reality. In particular, sensory impressions were hard to simulate, even though they have been found essential for assessing a major incident situation[17]. It is not known if simulated patients with moulage were used in the preparations or not, but such simulations might add more sensory impressions than for example table top training. Neither the skill to improvise was sufficiently covered in the preparations. Since improvisation is often used in emergency management[16,18], its value needs to be further highlighted in the preparations.

The structure and method used for major incident management rely on a command and control paradigm aimed at controlling the situation. This type of methodology has long been considered the 'gold standard' for managing such incidents. However, this approach has been criticized for being unrealistic because it is seldom possible to control major incidents to such extent that the models suggest[19]. As found in this study, the assumption of being able to control a major incident in all aspects might cause doubts in one´s capacity and

For peer review only - http://bmjopen.bmj.com/site/about/guidelines.xhtml

feelings of being exposed and vulnerable if there is a failure to gain control of an authentic incident. Such experiences have been described previously by other medical professionals when faced with disasters[20-22]. Therefore, it would appear important to reflect on how to balance striving to control and accepting uncontrollable dimensions of a major incident in the preparations.

One question that caused a burden to the commanders was the responsibility for safety and security of colleagues. The same issue has been described among prehospital incident commanders during tunnel incidents[23] and terrorist attacks[17]. During the interviews, reflections about acceptable levels of risks, formal and moral obligations, legal and professional responsibilities of ambulance personnel in general were raised, as well as such dilemmas in the specific context of a major incident. It became clear that these discussions were an important part of one's professional identity but also the mental preparedness for managing major incidents.

Managing stress was another challenge raised by the participants. Perceived stress is often a reasonable result of the demands to manage a large amount of uncertainty[24]. However, acting in an unfamiliar position, being witness to a large amount of human suffering, managing safety and security issues and high expectations both from oneself and others were also mentioned as stressors. It has been suggested that ambulance personnel need to develop strong self-resilient strategies to manage the stress inherent in both everyday work and critical incidents[25]. This study has shown that acting as a major incident commander adds several dimensions of stress compared to everyday situations. Stress management and resources to stay resilient during and after stressful and potentially traumatic events is not only a personally capacity—it is highly dependent on organizational support[26]. Therefore, stress management strategies should be seen as an essential skill that needs to be addressed in the preparations to enhance a resilient response to major incidents.

Professional experience was found to mediate some of the gaps between preparations and the authentic experiences. Since few incident commanders will have the possibility to gain a rich experience from authentic major incidents, trainings and other kinds of preparations is needed. The evaluation and feedback to participants in major incident trainings are often informal[27] or, as stated by the participants, focused on errors and shortcomings. By focusing on positive post-exercise debriefings[27] and by stimulating self-reflection during trainings[28] professional experience and self-confidence can be better enhanced. In addition, it can be suggested that learning debriefings or other feedback sessions focus not only on technical procedures and skills, but also on interpersonal and personal competences such as leadership, communication skills, and stress management. As suggested by participants in this study, sharing authentic experiences can also enrich personal preparations. Also, new technology and new ways of simulation might open up new possibilities to train and prepare for more aspect than the traditional major incident and disaster exercises[29].

Several suggestions on how to minimize the gap between preparations and authentic major incidents has been reported in this study. However, how to design and conduct effective educational sessions and training exercises and what methods should be used to develop technical skills and build the necessary professional and personal preparedness is a challenge that need to be further investigated.

Limitations
As in all qualitative studies, the results of this study rely on a sample of experiences gained

For peer review only - http://bmjopen.bmj.com/site/about/guidelines.xhtml

from personal experiences. The two-session approach[13] used was found to stimulate a deeper reflection and rich material to analyse. The second interview sessions were used to follow up on the first ones and were therefore in general shorter than these.
Some participants did not choose to participate in the second interview, but agreed to be included in the study based on their first interview session. The discussion during the analysis between all three authors provided a broad basis for the analysis, enriched by different professional backgrounds covering among other things disaster medicine, and crises management, health professions education and professional development.

CONCLUSIONS

Current major incident training prepares somewhat for actual major incident management. To cover for existing gaps between preparations and authentic experiences, future educational sessions and training exercises should reflect the complexity of a real major incident and stimulate the personal preparations to a larger extent. In addition to the development of technical skills and personal preparations, major incident preparations should contribute to building self-knowledge and professional self-confidence to act as a prehospital incident commander.

Funding

This research received no specific grant from any funding agency in the public, commercial or not-for-profit sectors.

DECLARATIONS

Ethics approval and consent to participate

The study was reviewed and approved by the Swedish Ethical Review Authority (ref id 2018:232).

Availability of data and materials

The data analysed during the current study are not publicly available due to Swedish laws on research ethics and the ethical approval.

Competing interests

The authors declare that they have no competing interests.

Author contributions

All authors participated in the planning and design of the study. KH conducted all interview and analysed the data, during discussions with SE and KB. KH was the major contributor in writing the manuscript, with the assistance of SE and KB. All authors read and approved the final manuscript.

REFERENCES

1. Lennquist Montán K, Hreckovski B, Dobson B, Örtenwall P, Montán C, Khorram-Manesh A, et al. Development and evaluation of a new simulation model for interactive training of the medical response to major incidents and disasters. *Eur J Trauma Emerg Surg* 2014;40(4):429-43.

For peer review only - http://bmjopen.bmj.com/site/about/guidelines.xhtml

2. Steigenberger N. Organizing for the Big One: A Review of Case Studies and a Research Agenda for Multi- Agency Disaster Response. *J. Contingencies Crisis Manag*. 2016; 24 (2): 60-72.
3. Hugelius K, Becker J, Adolfsson A. Five Challenges When Managing Mass Casualty or Disaster Situations: A Review Study. *Int J Environ Res Public Health*. 2020;17(9):E3068.
4. Rimstad R, Braut, GS. Literature review on medical incident commander. *Prehosp Disaster Med*. 2015;30(2):205-15.
5. Campion EM, Juillard C, Knudson MM, Dicker R, Cohen MJ, Mackersie R, et al. Reconsidering the Resources Needed for Multiple Casualty Events: Lessons Learned From the Crash of Asiana Airlines Flight 214. *JAMA surgery*. 2016;151(6):512-7.
6. Labrague LJ, Hammad K, Gloe DS, McEnroe-Petitte DM, Fronda DC, Obeidat AA, et al. Disaster preparedness among nurses: a systematic review of literature. *Int Nurs Rev*. 2018;65(01):41-53.
7. Hsu EB, Thomas TL, Bass EB, Whyne D, Kelen GD, Green GB. Healthcare worker competencies for disaster training. *BMC Med Educ*. 2006;6:19.
8. Perry RW, Peterson DM. Disaster Exercise Outcomes for Professional Emergency Personnel and Citizen Volunteers. *J. Contingencies Crisis Manag*. 2004;12(1):64-75.
9. Williams J, Nocera M, Casteel C. The Effectivness of Disaster Training for Health Care Workers: A Systematic Review. *Ann Emerg Med*. 2008;52(3):211-22.
10. Andersson A. In case of emergency. *Collaboration exercises at the bounderis between emergency service organization*. PhD Thesis. University West; Nb 8, Sweden. 2016
11. Gowing JR, Walker KN, Elmer SL, Cummings EA. Disaster Preparedness among Health Professionals and Support Staff: What is Effective? An Integrative Literature Review. *Prehosp Disaster Med*. 2017;32(3):321-8.
12. Legemaate GGA, Burkle F, Bierens JJLM. The Evaluation of Reseach Methods during Disaster Exercises: Applicability for Improving Disatser Health Management. *Prehosp Disaster Med*. 2012;27(1):18-25.
13. Read BL. Serial Interviews: When and Why to Talk to Someone More Than Once. *Int. J. Qual. Methods*. 2018; 17 (1): 1-10.
14. Arnberg F, Michel PO, Johannesson KB. Properties of Swedish Posttraumatic Stress Measures after a Disaster. *J Anxiety Disord*. 2014;28(4):402-9.
15. Braun V, Clarke, V. Using thematic analysis in psychology. *Qual Res Psychol*. 2006;3 (2):77-101.
16. Schakel JK, Wolbers, J. To the edge and beyond: How fast-response organizations adapt in rapidly changing crisis situations. *Hum Relat*. 2019:1-32.
17. Rimstad R, Sollid SJ. A retrospective observational study of medical incident command and decision-making in the 2011 Oslo bombing. *Int J Emerg Med*. 2015;4(8):4.
18. Brandrud AS BM, Brattebø G, Pedersen MJ, Håpnes K, Møller K, Bjorge T, Nyen B, Strauman L, Schreiner A, Haldorsen GS, Bergli M, Nelson E, Morgan TS, Hjortdahl P. Local emergency medical response after a terrorist attack in Norway: a qualitative study. *BMJ Qual Saf*. 2017;26(10):806-816.
19. Boersma F, Ferguson, P., Groeneweegen, J., Wolbers, J. *Beyond the Myth of Control: toward network switching in disaster management*. Proceedings of the 11th International ISCRAM Conference – University Park, May 2014. Pennsylvania, USA; 2014.
20. Hugelius K, Adolfsson A, Ortenwall P, Gifford M. Being both helpers and victims; Health professionals' experiences of working during a natural disaster. *Prehosp Disaster Med*. 2017;32(2):117-23.

For peer review only - http://bmjopen.bmj.com/site/about/guidelines.xhtml

21. Sloand E, Ho G, Kub J. Experiences of nurse volunteers in Haiti after the 2010 earthquake. *Res Theory Nurs Pract*. 2013;27(3):193-213.
22. Geisz-Everson MA, Dodd-McCue D, Bennett M. Shared Experiences of CRNAs Who Were on Duty in New Orleans During Hurricane Katrina. *AANA journal*. 2012;80(3):205.12.
23. Hylander J, Saveman BI, Björnstig U, Gyllencreutz L. Prehospital management provided by medical on-scene commanders in tunnel incidents in Oslo, Norway - an interview study. *Scand J Trauma Resusc Emerg Med*. 2019;27(1):78.
24. Peters A, McEwen BS, Friston K. Uncertainty and stress: Why it causes diseases and how it is mastered by the brain. *Prog Neurobiol*. 2017;156:164-88.
25. Clompus SR, Albarran JW. Exploring the nature of resilience in paramedic practice: A psycho-social study. *Int Emerg Nurs*. 2016;28:1-7.
26. Southwick SM, Charney DS. Resilience. *The Science of Mastering Life´s Greatest Challanges*. Second ed. New York: Cambridge University Press; 2018.
27. Skryabina E, Riley P, Reedy G, Amlôt R. A scoping review of evaluation methods for health emergency preparedness exercises. *Am J Disaster Med*. 2018;13(2):107-27.
28. Hermelin J, Bengtsson K, Woltjer R, Trnka J, Thorstensson M, Pettersson J, et al. Operationalising resilience for disaster medicine practitioners: capability development through training, simulation and reflection. Cogn Technol Work. 2020;22(3):667-83.
29. Cicero MX, Whitfill T, Walsh B, Diaz MC, Arteaga G, Scherzer DJ, Goldberg S, Madhok M, Bowen A, Paesano G, Redlener M, Munjal K, Kessler D, Auerbach M. 60 Seconds to Survival: A Multisite Study of a Screen-based Simulation to Improve Prehospital Providers Disaster Triage Skills. *AEM Educ Train*. 2018 Jan 31;2(2):100-106.

For peer review only

For peer review only - http://bmjopen.bmj.com/site/about/guidelines.xhtml

Standards for Reporting Qualitative Research (SRQR)*

http://www.equator-network.org/reporting-guidelines/srqr/

Page/line no(s).

Title and abstract

**Title** - Concise description of the nature and topic of the study Identifying the study as qualitative or indicating the approach (e.g., ethnography, grounded theory) or data collection methods (e.g., interview, focus group) is recommended	1
**Abstract** - Summary of key elements of the study using the abstract format of the intended publication; typically includes background, purpose, methods, results, and conclusions	1

Introduction

**Problem formulation** - Description and significance of the problem/phenomenon studied; review of relevant theory and empirical work; problem statement	2
**Purpose or research question** - Purpose of the study and specific objectives or questions	2

Methods

**Qualitative approach and research paradigm** - Qualitative approach (e.g., ethnography, grounded theory, case study, phenomenology, narrative research) and guiding theory if appropriate; identifying the research paradigm (e.g., postpositivist, constructivist/ interpretivist) is also recommended; rationale**	2
**Researcher characteristics and reflexivity** - Researchers' characteristics that may influence the research, including personal attributes, qualifications/experience, relationship with participants, assumptions, and/or presuppositions; potential or actual interaction between researchers' characteristics and the research questions, approach, methods, results, and/or transferability	3+11
**Context** - Setting/site and salient contextual factors; rationale**	2
**Sampling strategy** - How and why research participants, documents, or events were selected; criteria for deciding when no further sampling was necessary (e.g., sampling saturation); rationale**	2,3
**Ethical issues pertaining to human subjects** - Documentation of approval by an appropriate ethics review board and participant consent, or explanation for lack thereof; other confidentiality and data security issues	3
**Data collection methods** - Types of data collected; details of data collection procedures including (as appropriate) start and stop dates of data collection and analysis, iterative process, triangulation of sources/methods, and modification of procedures in response to evolving study findings; rationale**	3

For peer review only

**Data collection instruments and technologies** - Description of instruments (e.g., interview guides, questionnaires) and devices (e.g., audio recorders) used for data collection; if/how the instrument(s) changed over the course of the study	3
**Units of study** - Number and relevant characteristics of participants, documents, or events included in the study; level of participation (could be reported in results)	3,4
**Data processing** - Methods for processing data prior to and during analysis, including transcription, data entry, data management and security, verification of data integrity, data coding, and anonymization/de-identification of excerpts	3
**Data analysis** - Process by which inferences, themes, etc., were identified and developed, including the researchers involved in data analysis; usually references a specific paradigm or approach; rationale**	3
**Techniques to enhance trustworthiness** - Techniques to enhance trustworthiness and credibility of data analysis (e.g., member checking, audit trail, triangulation); rationale**	3

Results/findings

**Synthesis and interpretation** - Main findings (e.g., interpretations, inferences, and themes); might include development of a theory or model, or integration with prior research or theory	4-9
**Links to empirical data** - Evidence (e.g., quotes, field notes, text excerpts, photographs) to substantiate analytic findings	4-9

Discussion

**Integration with prior work, implications, transferability, and contribution(s) to the field -** Short summary of main findings; explanation of how findings and conclusions connect to, support, elaborate on, or challenge conclusions of earlier scholarship; discussion of scope of application/generalizability; identification of unique contribution(s) to scholarship in a discipline or field	9-10
**Limitations** - Trustworthiness and limitations of findings	11

Other

**Conflicts of interest** - Potential sources of influence or perceived influence on study conduct and conclusions; how these were managed	11
**Funding** - Sources of funding and other support; role of funders in data collection, interpretation, and reporting	11

*The authors created the SRQR by searching the literature to identify guidelines, reporting standards, and critical appraisal criteria for qualitative research; reviewing the reference lists of retrieved sources; and contacting experts to gain feedback. The SRQR aims to improve the transparency of all aspects of qualitative research by providing clear standards for reporting qualitative research.

For peer review only

**The rationale should briefly discuss the justification for choosing that theory, approach, method, or technique rather than other options available, the assumptions and limitations implicit in those choices, and how those choices influence study conclusions and transferability. As appropriate, the rationale for several items might be discussed together.

Reference:

O'Brien BC, Harris IB, Beckman TJ, Reed DA, Cook DA. **Standards for reporting qualitative research: a synthesis of recommendations.** *Academic Medicine*, Vol. 89, No. 9 / Sept 2014 DOI: 10.1097/ACM.0000000000000388

For peer review only

For peer review only - http://bmjopen.bmj.com/site/about/guidelines.xhtml

BMJ Open

Prehospital major incident management: How do training and real-life situations relate? A qualitative study.

Journal:	*BMJ Open*
Manuscript ID	bmjopen-2021-048792.R2
Article Type:	Original research
Date Submitted by the Author:	03-Sep-2021
Complete List of Authors:	Hugelius, Karin; Orebro University, Faculty of Medicine and Health<br>Edelbring, Samuel; Örebro University, Faculty of Medicine and Health<br>Blomberg, Karin; Örebro University, Faculty of Medicine and Health
**Primary Subject Heading**:	Emergency medicine
Secondary Subject Heading:	Emergency medicine
Keywords:	ACCIDENT & EMERGENCY MEDICINE, TRAUMA MANAGEMENT, HEALTH SERVICES ADMINISTRATION & MANAGEMENT

SCHOLARONE™
Manuscripts

I, the Submitting Author has the right to grant and does grant on behalf of all authors of the Work (as defined in the below author licence), an exclusive licence and/or a non-exclusive licence for contributions from authors who are: i) UK Crown employees; ii) where BMJ has agreed a CC-BY licence shall apply, and/or iii) in accordance with the terms applicable for US Federal Government officers or employees acting as part of their official duties; on a worldwide, perpetual, irrevocable, royalty-free basis to BMJ Publishing Group Ltd ("BMJ") its licensees and where the relevant Journal is co-owned by BMJ to the co-owners of the Journal, to publish the Work in this journal and any other BMJ products and to exploit all rights, as set out in our licence.

The Submitting Author accepts and understands that any supply made under these terms is made by BMJ to the Submitting Author unless you are acting as an employee on behalf of your employer or a postgraduate student of an affiliated institution which is paying any applicable article publishing charge ("APC") for Open Access articles. Where the Submitting Author wishes to make the Work available on an Open Access basis (and intends to pay the relevant APC), the terms of reuse of such Open Access shall be governed by a Creative Commons licence – details of these licences and which Creative Commons licence will apply to this Work are set out in our licence referred to above.

Other than as permitted in any relevant BMJ Author's Self Archiving Policies, I confirm this Work has not been accepted for publication elsewhere, is not being considered for publication elsewhere and does not duplicate material already published. I confirm all authors consent to publication of this Work and authorise the granting of this licence.

For peer review only

Prehospital major incident management: How do training and real-life situations relate? A qualitative study.

Karin Hugelius[1], Samuel Edelbring[1], Karin Blomberg[1]

1. Faculty of Medicine and Health, Örebro University, Örebro, Sweden.

Corresponding author:

Karin Hugelius

E-mail: karin.hugelius@oru.se

Address: Örebro University, 70182 Örebro, Sweden

Word count: 7143

For peer review only

Prehospital major incident management: How do training and real-life situations relate? A qualitative study.

ABSTRACT

Objective: To explore the relationship between preparations and real-life experiences among prehospital major incident commanders.

Design: An explorative, qualitative design was used.

Setting: Prehospital major incidents in Sweden. Data were collected between December 2019 and August 2020.

Participants: Prehospital major incident commanders (n = 15) with real-life experiences from major events, such as fires, bus accidents, a bridge collapse and terrorist attacks, were included. All but one had participated in two-day training focusing on the prehospital management of major incidents. In addition, about half of the participants had participated in simulation exercises, academic courses and other training in the management of major incidents.

Methods: Data from two session individual interviews were analysed using inductive thematic analysis.

Results: The conformity between real-life major incidents and preparations was good regarding prehospital major incident commanders' knowledge of the operational procedures applied in major incidents. However, the preparations did not allow for the complexities and endurance strategies required in real-life incidents. Personal preparations, such as mental preparedness or stress management, were not sufficiently covered in the preparations. To some extent, professional experience (such as training) could compensate for the lack of formal preparations.

Conclusions: This study identified perceived gaps between preparations and real-life experiences of being a prehospital major incident commander. To minimize the gaps between demands and expectations on perceived control and to better prepare individuals for being prehospital major incident commanders, the trainings and other preparations should reflect complexities of real-life incidents. Preparations should develop both technical skills required, such principles and methodology used, and personal preparedness. Personal preparations should include improving one's mental preparedness, self-knowledge and professional self-confidence required to successfully act as a prehospital incident commander. Since little is known about what pedagogical methods that should be used to enhance this, further research is needed.

Keywords: major incidents, prehospital management, disaster preparation, training, ambulance personnel, demands, control, support model

Strengths and limitations of this study

For peer review only - http://bmjopen.bmj.com/site/about/guidelines.xhtml

- The two-session approach used for the interviews stimulated a deeper reflection and rich material to analyse.
- The experiences from many different major incidents were gathered, thus providing a broad perspective on prehospital incident management.
- The multiprofessional background within the research team added different perspectives to the interpretation of the results.
- The limited number of study participants (n = 15) and the Swedish setting can impact the transferability of the results.
- Some interviews were conducted face-to-face, and others were conducted by phone. This may have influenced the sharing of experiences during the interviews.

For peer review only

BACKGROUND

Major incidents are defined as situations where available resources are insufficient for the immediate need of medical care.[1] Moreover, these challenging situations are often characterised by high levels of uncertainty, dynamic development and time critical decision-making.[2,3] The prehospital management of major incidents is an essential part of the overall medical response that requires specific skills and arrangements, such as an effective incident command.[4] However, structures in on-scene management vary around the world. In Sweden, at least one nurse, often specialised in prehospital emergency care, is on duty in each emergency ambulance. In the case of a major incident or disaster event, the first ambulance personnel that arrive at a scene will adopt the positions of the prehospital incident commander and medical officer in charge. These two positions may be carried out by either one or two persons. These positions ensure overall responsibility and have a mandate to lead and organise all aspects of the prehospital medical response, including overall decisions, priorities and requests, distributing prehospital resources and collaborating with relevant partners on site. In this study, the prehospital incident commander and prehospital medical officer in charge were studied without making any distinction between them; the term prehospital incident commander[4,5] will be used to cover both positions.

Since major incidents are relatively rare, many ambulance personnel do not have the opportunity to obtain extensive professional experience from such events. Therefore, training programmes and exercises are traditionally used to prepare responders.[6,7] Training exercises provide an opportunity to improve individual and team efforts, as well as the decision-making process, test technical equipment and working methods and increase awareness about the complexity of disaster situations.[7,8] To ensure that ambulance personnel are sufficiently prepared to adopt the role of a prehospital major incident commander, such knowledge is essential. Despite the long history and worldwide use of educational sessions and exercises to prepare for real major incidents or disasters, little is actually known about their effects.[6,9] Furthermore, the relationship between preparations and reality is an unexplored area.[10-12]

Therefore, the aim of this study was to explore the relationship between preparations and real-life experiences among prehospital major incident commanders.

METHODS

For peer review only - http://bmjopen.bmj.com/site/about/guidelines.xhtml

An explorative, qualitative design was used to gain insight into prehospital major incident commanders' experiences of major incidents in relation to previous preparations.

Study participants and recruitment

Swedish ambulance personnel who had experience being prehospital major incident commanders and/or prehospital medical officers during a least one major incident (defined as an incident that had led to a heightened level of response within the healthcare system) during the previous five years were included in the study. The study participants were recruited between December 2019 and August 2020 via written invitations sent to ambulance services where major incidents had occurred during the last five years. In addition, the same invitation was posted on social media groups and forums aimed specifically at Swedish ambulance personnel. If interested in participating in the study, the participants were asked to access a web page for information on the study; here, the participants could sign up for the study. Subsequently, the first author (KH) contacted the participants to provide further information on the study and to set a suitable time for the first interviews. All prehospital major incident commanders who volunteered to participate in the study matched the criteria and were thus included.

Data collection procedure

A total of 15 Swedish prehospital major incident commanders were interviewed in 27 individual interviews, conducted in two session interviews.[13] The first interview was followed by a second interview within two weeks. This approach was used to ensure that reflections and thoughts that had been raised during the first interview were followed up in the second to ensure rich data.[13] Three of the study participants declined a second interview due to personal reasons (such as planned holidays). A semi-structured interview guide was used, based on previous research, and had been developed in discussion by all the authors. The questions used in the first set of interviews covered demographic information, previous educational sessions and training exercises in managing major incidents and questions regarding the participants' experiences from such incidents. In the second set of interviews, which followed up on the first interview, the study participants were asked if they had any additional thoughts or experiences that had not been mentioned in the first set of interviews. The interview guide was piloted with two prospective participants, after which only small adjustments were made. Therefore, the pilot interviews were included in the analysis. In addition, all study participants were asked to complete the Swedish civilian version of the posttraumatic checklist, with a cut-off value of 40 used as an indicator for recommending professional assessment of post-traumatic stress.[14] The first and second session interviews lasted between 24 and 89 min (median 53 min) and 5 and 22 min (median 12 min), respectively. The total interview time was approximately 16 hours.

All interviews were conducted by the first author (KH), either by telephone (*n* = 13) or face-to-face (*n* = 2), depending on geographical distance and the COVID-19 pandemic. Face-to-face interviews were conducted in privacy at the participants' workplace. Full study information was provided before the interviews started, and digital informed consent was obtained from the participants before the first interview. The study was reviewed and approved by the Swedish Ethical Review Authority (ref id 2018:232).

Analysis

For peer review only - http://bmjopen.bmj.com/site/about/guidelines.xhtml

The interviews were transcribed in full by a professional transcriber and subsequently checked by the interviewer. An inductive thematic analysis[14] approach was used to identify the skills and experiences requested and used in real-life incidents, and to relate the preparations to those. First, all interviews were read through to gain a sense of the complete data. Features pertaining to the study purpose were then marked, extracted and coded. Initially, main themes were identified. Within these, themes and subthemes were then formed from the codes based on similarities and differences between them. Thematic maps[15] were then used to analyse the relations between the main perspectives, themes and subthemes. All themes and subthemes were labelled and described. Finally, a comprehensive summary of the results, showing the relations between the themes, was formulated. Quotations were identified and used to illustrate the findings and increase trustworthiness. The analysis was initially performed by the first author (KH), then reviewed and discussed with the other authors (SE and KB).

Patient and public involvement

Given the focus on the experiences and training of ambulance personnel, patients and the public were not involved in the design, data collection or data analysis.

RESULTS

Of the 15 prehospital major incident commanders that were interviewed, 10 were male and 5 were female. Their ages varied from 29 to 62 years, with a median of 38 years. Their professions were as follows: ambulance nurses with a specialisation in ambulance care (n = 7), nurses with a specialisation in anaesthesia care (n = 4) and general nurses (n = 4). They had all been deployed within ambulance services between 1 and 39 years (median of 18 years). All except one was trained in prehospital major incidents management by participating in a standardised two-day course consisting of both theoretical and practical training. The theoretical training covered legal aspects, reporting procedures and mass casualty triage, and the practical training consisted of tabletop exercises on whiteboards using the Emergo Train System®[16] and one or two simulation exercises, such as traffic accidents. Other preparations varied from no other training (except for the above-mentioned two-day training) (n = 8) to disaster simulation exercises covering up to four hours (n = 5), major incident training as part of being a voluntary staff member within a non-governmental organisation (n = 2), academic courses on disaster medicine (n = 1) or major incident exercises within the military services (n = 1). None of the participants had participated in simulations covering more than four hours, neither in digital nor other kinds of simulation-based activities.

The patients' experiences in major incidents, as revealed in the interviews, included the following: a bridge collapse; car, bus or train accidents; a vehicle-ramming attack; an aircraft crash; a shooting; an explosion; a chemical incident; and a fire in an elderly home care centre. Some participants had experienced several major incidents. According to the civilian posttraumatic checklist, none of the participants exhibited any signs of being traumatised by their experiences.

The following three main themes emerged: *skills and experiences are needed to manage major incidents, skills significantly covered in preparations* and *gaps in preparations in relation to the demands of real-life major incidents*. Six additional themes were identified:

For peer review only - http://bmjopen.bmj.com/site/about/guidelines.xhtml

manage the response, being in charge, frames for management, operational procedures, personal preparedness and *professional experience* (see Table 1).

Table 1. Overview of main themes, themes and subthemes

**Main themes**	**Skills and experiences needed to manage major incidents**		**Skills significantly covered in preparations**	**Gaps in preparations in relation to the demands of real-life major incidents**		
**Themes**	Manage the response	Being in charge	Frames for management	Operational procedures	Personal processes	Lack of professional experience
**Sub-themes**	To use a certain methodology	To feel lonely and exposed	Principles and methodology	Manage the complexity	Mental preparedness	Interpretation of the situation
	To grasp the situation	To be responsible for others		Manage endurance	Ethical dilemmas	Mediating stress
	To make decisions	To manage stress			Self-knowledge	
	To improvise	To trust one's capability				

Skills and experiences needed to manage major incidents

This main theme describes the experiences of being a prehospital major incident commander in real-life events and the skills needed for such management.

Manage the response

The first information presented from the dispatch centre to the incident commanders about the incident was vague, and they had limited information on the extent and character of the situation. Therefore, few of them were not mentally prepared to deal with a major incident when arriving at the scene.

To gain control of the situation and plan for dynamic development, the incident commanders strived to obtain an overview of the actual event as soon as they arrived at the scene. The importance of stopping and thinking, taking a breath and assimilating the scene instead of rushing into it was strongly emphasised:

> *Stop and ... and think. There is always time to stop and think about the whole thing before you get into such a situation. Even if it is chaotic when you arrive, it is worth taking these extra ... to use 20–30 seconds and try to get a picture of the situation and the victims. You should try to make a plan before entering the scene.* (Participant 5, referring to general experiences of being a prehospital major incident commander)

Several participants referred to a structure and methodology they had been taught to organise their response. Thus, sufficient knowledge of the principles and structure used to manage major incidents was necessary. Examples of such principles were mass casualty

For peer review only - http://bmjopen.bmj.com/site/about/guidelines.xhtml

triage or reporting algorithms. Maintaining the agreed methodology, especially during the beginning of the response, was emphasised. However, details regarding the methodology and concepts differed from region to region, which is why local knowledge on, for example, when to report what and to whom, is needed.

Sensory impressions (such as vision, hearing, sight and smell), combined with professional experience and a 'gut feeling' based on knowledge and intuition, were used to interpret the situations and plan for consequences and possible developments. In this process, previous experiences from both real-life situations and training could contribute.

> *I spontaneously felt in my body that I recognized this as a very difficult, major incident and at the same time I was somehow mentally prepared that something more would happen. So, I started to look for more injured. My intuition told me that there were more [injured] to find.* (Participant 2, referring to a traffic accident)

Since conditions change every minute, the analysis of consequences and potential development of an incident was a continually ongoing process. Consequently, the development of these situations is hard to predict, meaning that incident commanders have to be constantly prepared to adapt their strategies and actions. Examples of unexpected conditions could be to find more victims, unexpected changes in patients' medical conditions, a sudden weather change or an escalating security situation. The incident commanders strive to be one step ahead and plan for different scenarios, using their professional experience and local knowledge to guide them:

> *The conditions changed immediately at the triage site when one of the patients had convulsions. From being up walking, he just collapsed and developed general convulsions. I had to change the plan and made the helicopter take him immediately…* (Participant 1, referring to a bridge collapse)

Several decisions had to be made, which in many cases had to be conducted quickly and based only on fragmented information. To make wise decisions, the incident commander requires good local knowledge (such as geography and logistical aspects). Knowledge of the capacity of nearby hospitals and personal knowledge of the ambulance personnel present at the scene facilitated the management and use of available resources. If local knowledge was lacking, the incident commanders risk making decisions with major consequences, such as making wrong or late decisions concerning the distribution of patients, possible locations for evacuation and other logistical questions.

Being in charge

Being an incident commander entails the organisation of the medical response, leading colleagues and making decisions that would greatly impact others. The mandate of being an incident commander, which was both formal and informal, implies a greater power and responsibility compared to their normal work. To be trusted and respected, the incident commanders strive to clearly demonstrate their capability to manage the situation. For example, they use calm body language, talk slowly and loudly and change their methods of communication to shorter and more concise information when compared to their everyday situations. Communication at the scene often relies on face-to-face communication, physical signals, such as a 'thumbs-up', or eye contact. Sometimes, according to other colleagues, the incident commander does not fulfil their obligations. At such times, colleagues can feel

For peer review only - http://bmjopen.bmj.com/site/about/guidelines.xhtml

frustrated, which in turn makes them feel obligated to make their own decisions to compensate for the perceived absence of management.

Most often, these decisions are time critical and could have life-changing consequences, which can cause added tension and ethical dilemmas. The decision-making was most often made by the incident commander on their own, and being aware that their decisions and actions could make the difference between life and death makes the incident commanders feel lonely and exposed:

> *We had no one to ask. It was just my colleague and myself. There was no one else. Everything depended on us. We are used to it, but it's a thing when you have so many people that are dependent on you… I think maybe you do not understand this until after quite a few years within this profession. What you as incident commander decide can have huge consequences for others. We make decisions that otherwise the most senior medical doctor inside the hospital makes. You need to understand that the responsibility is huge.* (Participant 12, referring to a bus- train crash)

Distanced support, such as from a remote senior advisor on commanding an incident, was not always perceived as supportive. If not present at the site, many of the impressions and complexities of situations may not be grasped, as it is difficult for someone not physically present to assimilate all the aspects of the situation. Technical means of communication (such as radios or mobile phones) are less supportive than expected, given the chaotic and often noisy environment. Another aspect of remote support was that it does not assist in the very early stages when many of the toughest decisions need to be made.

Maintaining the role of a manager also implies being responsible for ambulance personnel. In particular, security decisions such as if (and when) to send colleagues into collapsed buildings, rubble or hostile environments stressed the incident commanders:

> *It is a dangerous world …. But if you have chosen to work within the ambulance services, then you need to have courage, because we are the last outpost in society in some way. Although it can be scary, and it is dangerous, we must face it; we are not protected and if you choose this profession then you have to be prepared for that as well. I think it belongs to the profession.* (Participant 9, referring to a terrorist attack)

It was emphasised that being among the ambulance personnel sometimes implies working in risky environments. However, a general lack of conformity regarding acceptable risks hampers the decision-making process and sometimes causes conflicts between the incident commander and ambulance personnel. The presence or perceived force in the stress reactions experiences by the prehospital major incident commanders, and how these reactions limit their cognitive ability surprised the incident commanders. For example, the capability to maintain time perception was lost by many of the incident commanders. Stress does not only affect the incident commanders, but all personnel present at the site. Sometimes, the incident commanders must adapt their plans and their leadership style to the reactions exhibited by other ambulance or rescue personnel:

> *To make wise decisions I must consider; do we have ambulance personnel on site who are in distress… so that they cannot handle it or… are my colleagues okay? That is also an important thing to consider when you make decisions.* (Participant 10, referring to a bus crash)

For peer review only - http://bmjopen.bmj.com/site/about/guidelines.xhtml

A certain level of self-trust is necessary to enable the management of their own feelings and reactions while simultaneously managing the complexity of these situations. If the incident commander feels safe, trusted and supported by their colleagues, they can focus more on the actual incident management and less on their own reactions and limitations.

Skills significantly covered in preparations

Some aspects were sufficiently covered in the preparations. The structure and principles, such as how to organise the response, nomenclature and reporting and triage systems, formed the basis for a structured methodology to manage major incidents:

> *The moments that I really benefitted from were that you know that there is a structure, and you know who to contact and what to report when.* (Participant 2, referring to traffic accident)

Being aware of the rules and regulations pertaining to the medical response and other stakeholders' mandates was necessary to enhance collaboration with those engaged stakeholders. Logistics, including the flow of patients and distribution of victims, are other issues that require specific knowledge. The sessions teaching such frameworks were mainly theoretical and were sometimes enacted in tabletop training. All these formal strategies were sufficiently covered in the training, forming a basis for the incident commander to rely on.

Gaps in preparations in relation to the demands of real-life major incidents

Within this theme, the gaps in preparations regarding the demands of managing a real-life event were described. The gaps found were related to operational procedures, personal processes and professional experiences.

Operational procedures

Real-life incidents are usually found to be more complex and harder to grasp than previous training sessions conveyed. Sensory impressions and timelines are especially hard to represent in simulations, and thus, training the use of intuition based on such impressions, or training stress reactions such as cognitive impairment, was difficult to obtain.

Collaboration with other actors at the scene (such as rescue services or the police) is necessary for the smooth management of the scene. This collaboration not only focuses on 'what to do' questions, such as methodological or strategic questions, but also on 'how to do it' questions, such as ethical considerations or leadership styles. These aspects were seldom discussed as part of the training sessions, causing a gap in the preparations. Most often, the training exercises focused on the first start-up phase, such as how to act when arriving at the scene. However, real-life major incidents last longer than any training or exercise.

> *In general, I think we often talk about the start-up phase, about what we should do when arriving at the scene. But it is just as important to step down and end the mission in a good way. And all in between… to make sure that the right patient comes to the right hospital, that all colleagues are okay, that we have control of the equipment and so, to make sure that the step down is smooth.... We never train that.* (Participant 13, referring to a bus crash)

The importance of preparing for the training of long-term management, stepping down and performing a closure of the site was emphasised.

For peer review only - http://bmjopen.bmj.com/site/about/guidelines.xhtml

Personal processes

While many processes on a personal level are activated during real major incidents, this did not occur during training or exercises. Sensory impressions, such as sights, sounds, smells or the general life and death awareness of actual victims compared to a manikin, were some of these. Such impressions increased the perceived stress in a way that was hard to attain in simulations:

> *…but when you stand there, there are hearing impressions and you see things…and you smell things… and it is a warm body you hold in your hands and you feel your own pulse when you get stressed in a completely different way than during exercises.* (Participant 11, referring to a terrorist attack)

Being mentally prepared is an essential part of the personal preparations required to hold the position of a prehospital major incident commander. Being mentally prepared mainly refers to a personal process of reflection on what it really means to be ambulance personnel in general and at a major incident in particular. Since the system implies that almost every ambulance personnel on duty could arrive at a major incident first and become the incident commander, this mental preparedness needs to be part of their everyday preparations when beginning their shift:

> *You are never prepared when you come to work in the morning to be standing in the middle of a bridge collapse with 10 to 15 injured five hours later. You are not personally prepared for that. But as a professional, I need to be mentally prepared and think it though beforehand. I have to think, "How would I act if this happened?" That's your own responsibility and a good way to prepare yourself.* (Participant 1, referring to general experiences from major incidents)

Being aware of one's own personal and professional qualities and limitations, and preparing to feel exposed, lonely and powerless, are also part of the mental preparation process. It was also stressed that preparing for such feelings involves listening and learning from others with experience of major incidents, in addition to using one's own imagination.

Self-knowledge, including being aware of how others perceive one's leadership and appearance, personal attributes and discussions on ethical dilemmas when managing real-life incidents are all highlighted as being absent from the training. Furthermore, the design and implementation of training affects personal preparations both positively and negatively. For instance, by acquiring a perceived feeling of having succeeded, rather than failing and learning from the mistakes, personal growth could be obtained during the training that could be used in real-life major incidents. By providing the chance to repeat one's performance until success was achieved, and by positive coaching and feedback during the training session, positive and increased self-esteem could be promoted. If the opportunity was provided to repeat the performance after feedback and in-depth reflections, a final feeling of having succeeded could be obtained. Such cognitive and physical memories could increase the possibility of acting correctly by instinct when faced with a similar challenge in the future.

Lack of professional experience

Since major incidents are unusual events, many years of deployment within the ambulance service is required to gain sufficient experience to manage a major incident. In many situations, the personnel in managing positions at major incidents could be quite new and

For peer review only - http://bmjopen.bmj.com/site/about/guidelines.xhtml

inexperienced. Professional experience from real-life major incidents could, to some extent, compensate for a lack of formal training and preparations. Having sufficient professional experience as ambulance personnel in an everyday perspective, specifically from acting as a prehospital medical commander in both minor and major incidents, is of great importance to be able to function in such a position during a major incident:

> *You need experience, long experience. And you have to be able to manage much more than what they say on the course ... that is not enough* […] *You need to take so much into consideration; geography, roles, laws and things like that. Then you need to be calm and confident in order to get people to act as you would like them to...so that they trust you* (Participant 8, referring to an explosion).

Professional experience adds value when analysing the potential development of a situation, when making decisions under pressure and for bearing the responsibility that comes with the role:

> *Afterwards I can feel like "I fixed this," so I feel more self-confident […] I actually do the right thing and think the right things in situations like this. It was very positive for me; it has made me feel more confident in my professional role…* (Participant 5, referring to a bus crash)

In addition, professional experience enriches self-trust and tolerance for one's own limitations and balances feelings of being exposed, which can make the incident commander more comfortable in the situation. Lacking professional experience has the opposite effect. Also, a lack of experience from major incidents and from working within ambulance services in general reduces the necessary ability of a commander in a major incident to tolerate and manage stress.

DISCUSSION

This study identified areas that need further attention and applied guidance on how to prepare prehospital major incident commanders more effectively in the future. The conformity between real-life major incidents and preparations was good regarding sufficient preparedness for operational procedures such as organisation, reporting structure and triage methods. However, the preparations did not prepare for the complexity and endurance strategies required in real-life major incidents. Incident commanders were not prepared for several personal processes, such as stress management, ethical dilemmas and the self-knowledge required to handle real-life major incident situations. To some extent, professional experience could compensate for a lack of training. However, incident managers requested further, and to some extent different, preparations to incorporate operational procedures, professional experiences and personal preparedness to match the complexity and demands of real-life major incidents.

The incident commanders described how they used information from many sources, in addition to sensory impressions and their own experience, to interpret the situation, predict development and make decisions. This process corresponds to the concept of situational awareness, as in the perception and understanding of the situation and its meaning.[17] Developing situational awareness is crucial for responding adequately to crises. However, this study demonstrated that this complex process was hard to grasp in the training and exercises, since these were not perceived as being as complex as reality. In particular, sensory impressions were hard to simulate, even though they have been found essential for

For peer review only - http://bmjopen.bmj.com/site/about/guidelines.xhtml

assessing a major incident situation.[18] It is not known if simulated patients during training were used in the preparations or not, but such simulations might add more sensory impressions other than, for example, tabletop trainings. Additionally, the skill to improvise was not sufficiently covered in the preparations. Since improvisation is often used in emergency management[17,19], its value needs to be further highlighted in the preparations.

The structure and method used for major incident management rely on a command and control paradigm aimed at controlling the situation. This type of methodology has long been considered the 'gold standard' for managing such incidents. However, this approach has been criticised for being unrealistic because it is seldom possible to control major incidents to such an extent, as the models suggest.[20] As found in this study, the assumption of being able to control a major incident in all aspects might cause doubts in one's capabilities, as well as feelings of being exposed and vulnerable if there is a failure to gain control of an incident. Such experiences have been previously described by other medical professionals when faced with disasters.[21-23] Therefore, it would appear important to reflect on how to balance the ability to control a situation and how to accept uncontrollable dimensions of a major incident in the preparations.

One question that caused a burden to the commanders pertained to the responsibility for the safety and security of colleagues. The same issue has been described among prehospital incident commanders during tunnel incidents[24] and terrorist attacks.[18] During the interviews, reflections about the acceptable levels of risks, formal and moral obligations, legal and professional responsibilities of ambulance personnel in general were raised, as well as dilemmas in the specific context of a major incident. It became clear that these discussions were an important part of one's professional identity and mental preparedness for managing major incidents.

Managing stress was another challenge raised by the participants. Since one of the characteristics of major incidents is a high level of uncertainty[2,3], and the essence of stress has been suggested to consist of uncertainty [25], this finding is not surprising. The job demand-control-support model[26] that has been widely used to explain work related stress illustrates the relation between demands, both quantitative, qualitative, and emotional, in relation to experience level of control and available support. Acting in an unfamiliar position, being witness to a large amount of human suffering and managing safety and security issues and high expectations both from oneself and others were experiences expressed by the prehospital major incident commanders. The combination of high demand and low control may lead to an increased risk of both physical and psychosocial health problems in both a short and long term perspective.[27] It has been suggested that incident managers' personnel need to develop strong self-resilient strategies to manage the stress inherent in both everyday work and critical incidents.[28] However, stress management and the resources needed to stay resilient during and after stressful and potentially traumatic events is not only a personal choice, but it is highly dependent on organisational support.[29] This statement is supported by the finding that the social support from colleagues and managers is an important "buffer" that may moderate the negative effects on perceived stress, mental health, and job strain[27]. Therefore, individual stress management strategies as well as a supportive climate within the working group should be seen as an essential preparation among prehospital major incident commanders to enhance a resilient response. Also, mental preparedness including ethical and moral reflections seems to be an essential component to minimize the gap between experiences and formal demands and experienced level of

For peer review only - http://bmjopen.bmj.com/site/about/guidelines.xhtml

control. However, the health consequences from being a prehospital incident commander and their relation to preparations is an area that remains unexplored, requiring further research interest.

Professional experience was found to mediate some of the gaps between preparations and real-life experiences. Since few incident commanders will have the possibility of gaining rich experience from real-life major incidents, training and other kinds of preparations are needed. The evaluation and feedback to participants in major incident training are often informal[30] or, as stated by the participants, focused on errors and shortcomings. By focusing on positive post-exercise debriefings[29] and by stimulating self-reflection during training,[31] professional experience and self-confidence can be better enhanced. In some organisations, video cameras or drone cameras are being used to document the scene and provide an overview of the situation. Such tools may add value to learning debriefings. In addition, it can be suggested that learning debriefings or other feedback sessions focus not only on technical procedures and skills, but also on interpersonal and personal competences, such as leadership, communication skills and stress management. As suggested by the participants in this study, sharing experiences from real major incidents can also enrich personal preparations and add several dimensions except from the technical or organizational aspects usually discussed in lesson learned sessions. Also, new technology and new simulations can open the door to new possibilities to train and prepare for a wider array of conditions than the traditional major incident and disaster exercises.[32] Since the knowledge on what particular exercise components that are vital for achieving learning in major incident or disaster trainings for health care professionals is still limited[33], this is a field requiring more scientific interest.

Limitations

As in all qualitative studies, the results of this study rely on a sample of personal experiences. The two-session approach[13] was found to stimulate a deeper reflection and provided rich material to analyse. The second interview sessions were used to follow up on the first ones and were therefore shorter. Some participants did not choose to participate in the second interview, but they agreed to be included in the study based on their first interview session. Another limitation was the lack of detailed information collected on of the participants´ preparations in terms of exact content of courses or trainings. However, this qualitative study does not aim to distinguish between specific trainings or preparations. The discussion during the analysis between all three authors provided a broad basis for the analysis, enriched by different professional backgrounds covering, among other things, disaster medicine, crises management, health professions education and professional development.

CONCLUSION

This study identified perceived gaps between preparations and real- life experiences of being a prehospital major incident commander. To minimize these gaps between demands and expectations on perceived control, and better prepare individuals for being prehospital major incident commanders, the trainings and other preparations should reflect complexities of real-life incidents. Preparations should promote and develop both technical skills required, such principles and methodology used in major incidents, and personal preparedness. Personal preparations should include improving one’s mental preparedness, self-knowledge and professional self-confidence required to successfully act as a prehospital

For peer review only - http://bmjopen.bmj.com/site/about/guidelines.xhtml

incident commander. However, the design and conduct of effective educational sessions and training exercises and what pedagogical methods should be used to develop both technical skills required and build the necessary professional and personal preparedness is still a challenge that needs to be further investigated. In addition, studies on the relation between preparations and health consequences from being a prehospital major incident commander is an area requiring further attention.

Funding
This research received no specific grant from any funding agency in the public, commercial or not-for-profit sectors.

DECLARATIONS

Ethics approval and consent to participate
The study was reviewed and approved by the Swedish Ethical Review Authority (ref id 2018:232).

Availability of data and materials
The data analysed during the current study are not publicly available due to Swedish laws on research ethics and the ethical approval provided by the Swedish Ethical Review Authority.

Competing interests
The authors declare that they have no competing interests.

Author contributions
All authors participated in the planning and design of this study. KH conducted all interviews and analysed the data during discussions with SE and KB. KH was the major contributor in writing the manuscript, with the assistance of SE and KB. All authors read and approved the final manuscript.

REFERENCES

1. Lennquist Montán K, Hreckovski B, Dobson B, Örtenwall P, Montán C, Khorram-Manesh A, et al. Development and evaluation of a new simulation model for inter
ive training of the medical response to major incidents and disasters. *Eur J Trauma Emerg Surg* 2014;40(4):429-43.
2. Steigenberger N. Organizing for the Big One: A Review of Case Studies and a Research Agenda for Multi- Agency Disaster Response. *J. Contingencies Crisis Manag*. 2016; 24 (2): 60-72.
3. Hugelius K, Becker J, Adolfsson A. Five Challenges When Managing Mass Casualty or Disaster Situations: A Review Study. *Int J Environ Res Public Health*. 2020;17(9):E3068.
4. Rimstad R, Braut, GS. Literature review on medical incident commander. *Prehosp Disaster Med*. 2015;30(2):205-15.
5. Campion EM, Juillard C, Knudson MM, Dicker R, Cohen MJ, Mackersie R, et al. Reconsidering the Resources Needed for Multiple Casualty Events: Lessons Learned From the Crash of Asiana Airlines Flight 214. *JAMA surgery*. 2016;151(6):512-7.
6. Labrague LJ, Hammad K, Gloe DS, McEnroe-Petitte DM, Fronda DC, Obeidat AA, et al. Disaster preparedness among nurses: a systematic review of literature. *Int Nurs Rev*. 2018;65(01):41-53.

For peer review only - http://bmjopen.bmj.com/site/about/guidelines.xhtml

7. Hsu EB, Thomas TL, Bass EB, Whyne D, Kelen GD, Green GB. Healthcare worker competencies for disaster training. *BMC Med Educ*. 2006;6:19.
8. Perry RW, Peterson DM. Disaster Exercise Outcomes for Professional Emergency Personnel and Citizen Volunteers. *J. Contingencies Crisis Manag*. 2004;12(1):64-75.
9. Williams J, Nocera M, Casteel C. The Effectivness of Disaster Training for Health Care Workers: A Systematic Review. *Ann Emerg Med*. 2008;52(3):211-22.
10. Andersson A. In case of emergency. *Collaboration exercises at the bounderis between emergency service organization*. PhD Thesis. University West; Nb 8, Sweden. 2016
11. Gowing JR, Walker KN, Elmer SL, Cummings EA. Disaster Preparedness among Health Professionals and Support Staff: What is Effective? An Integrative Literature Review. *Prehosp Disaster Med*. 2017;32(3):321-8.
12. Legemaate GGA, Burkle F, Bierens JJLM. The Evaluation of Reseach Methods during Disaster Exercises: Applicability for Improving Disatser Health Management. *Prehosp Disaster Med*. 2012;27(1):18-25.
13. Read BL. Serial Interviews: When and Why to Talk to Someone More Than Once. *Int. J. Qual. Methods*. 2018; 17 (1): 1-10.
14. Arnberg F, Michel PO, Johannesson KB. Properties of Swedish Posttraumatic Stress Measures after a Disaster. *J Anxiety Disord*. 2014;28(4):402-9.
15. Braun V, Clarke, V. Using thematic analysis in psychology. *Qual Res Psychol*. 2006;3 (2):77-101.
16. Emergo Train System®. 2016. Avalible at: https://www.emergotrain.com/. Accessed: August 01, 2021.
17. Schakel JK, Wolbers, J. To the edge and beyond: How fast-response organizations adapt in rapidly changing crisis situations. *Hum Relat*. 2019:1-32.
18. Rimstad R, Sollid SJ. A retrospective observational study of medical incident command and decision-making in the 2011 Oslo bombing. *Int J Emerg Med*. 2015;4(8):4.
19. Brandrud AS BM, Brattebø G, Pedersen MJ, Håpnes K, Møller K, Bjorge T, Nyen B, Strauman L, Schreiner A, Haldorsen GS, Bergli M, Nelson E, Morgan TS, Hjortdahl P. Local emergency medical response after a terrorist attack in Norway: a qualitative study. *BMJ Qual Saf*. 2017;26(10):806-816.
20. Boersma F, Ferguson, P., Groeneweegen, J., Wolbers, J. *Beyond the Myth of Control: toward network switching in disaster management*. Proceedings of the 11th International ISCRAM Conference – University Park, May 2014. Pennsylvania, USA; 2014.
21. Hugelius K, Adolfsson A, Ortenwall P, Gifford M. Being both helpers and victims; Health professionals' experiences of working during a natural disaster. *Prehosp Disaster Med*. 2017;32(2):117-23.
22. Sloand E, Ho G, Kub J. Experiences of nurse volunteers in Haiti after the 2010 earthquake. *Res Theory Nurs Pract*. 2013;27(3):193-213.
23. Geisz-Everson MA, Dodd-McCue D, Bennett M. Shared Experiences of CRNAs Who Were on Duty in New Orleans During Hurricane Katrina. *AANA journal*. 2012;80(3):205.12.
24. Hylander J, Saveman BI, Björnstig U, Gyllencreutz L. Prehospital management provided by medical on-scene commanders in tunnel incidents in Oslo, Norway - an interview study. *Scand J Trauma Resusc Emerg Med*. 2019;27(1):78.
25. Peters A, McEwen BS, Friston K. Uncertainty and stress: Why it causes diseases and how it is mastered by the brain. *Prog Neurobiol*. 2017;156:164-88.
26. Karasek R, Theorell T. *Stress healthy work: productivity and the reconstructions of work life* (1990) New York, USA: Basic Books.

For peer review only - http://bmjopen.bmj.com/site/about/guidelines.xhtml

27. Pan, K. Y., Xu, W., Mangialasche, F., Wang, R., Dekhtyar, S., Calderón-Larrañaga, A., . . . Wang, H. X. Psychosocial working conditions, trajectories of disability, and the mediating role of cognitive decline and chronic diseases: A population-based cohort study. *PLoS Med*. 2019; 16(9), e1002899.
28. Clompus SR, Albarran JW. Exploring the nature of resilience in paramedic practice: A psycho-social study. *Int Emerg Nurs*. 2016;28:1-7.
29. Southwick SM, Charney DS. Resilience. *The Science of Mastering Life´s Greatest Challanges*. Second ed. New York: Cambridge University Press; 2018.
30. Skryabina E, Riley P, Reedy G, Amlôt R. A scoping review of evaluation methods for health emergency preparedness exercises. *Am J Disaster Med*. 2018;13(2):107-27.
31. Hermelin J, Bengtsson K, Woltjer R, Trnka J, Thorstensson M, Pettersson J, et al. Operationalising resilience for disaster medicine practitioners: capability development through training, simulation and reflection. Cogn Technol Work. 2020;22(3):667-83.
32. Cicero MX, Whitfill T, Walsh B, Diaz MC, Arteaga G, Scherzer DJ, Goldberg S, Madhok M, Bowen A, Paesano G, Redlener M, Munjal K, Kessler D, Auerbach M. 60 Seconds to Survival: A Multisite Study of a Screen-based Simulation to Improve Prehospital Providers Disaster Triage Skills. *AEM Educ Train*. 2018 Jan 31;2(2):100-106.
33. Waring, S, Skryabina, E, Goodwin, D, Lino, D, & Amlôt, R. What components of emergency preparedness exercises improve healthcare practitioners' emergency response learning? *Int. J. Disaster Risk Reduct*. 2021;62, 102357.

For peer review only

For peer review only - http://bmjopen.bmj.com/site/about/guidelines.xhtml

Standards for Reporting Qualitative Research (SRQR)*

http://www.equator-network.org/reporting-guidelines/srqr/

Title and abstract

	Page/line no(s).
**Title** - Concise description of the nature and topic of the study Identifying the study as qualitative or indicating the approach (e.g., ethnography, grounded theory) or data collection methods (e.g., interview, focus group) is recommended	1
**Abstract** - Summary of key elements of the study using the abstract format of the intended publication; typically includes background, purpose, methods, results, and conclusions	1

Introduction

**Problem formulation** - Description and significance of the problem/phenomenon studied; review of relevant theory and empirical work; problem statement	2
**Purpose or research question** - Purpose of the study and specific objectives or questions	2

Methods

**Qualitative approach and research paradigm** - Qualitative approach (e.g., ethnography, grounded theory, case study, phenomenology, narrative research) and guiding theory if appropriate; identifying the research paradigm (e.g., postpositivist, constructivist/ interpretivist) is also recommended; rationale**	2
**Researcher characteristics and reflexivity** - Researchers' characteristics that may influence the research, including personal attributes, qualifications/experience, relationship with participants, assumptions, and/or presuppositions; potential or actual interaction between researchers' characteristics and the research questions, approach, methods, results, and/or transferability	3+11
**Context** - Setting/site and salient contextual factors; rationale**	2
**Sampling strategy** - How and why research participants, documents, or events were selected; criteria for deciding when no further sampling was necessary (e.g., sampling saturation); rationale**	2,3
**Ethical issues pertaining to human subjects** - Documentation of approval by an appropriate ethics review board and participant consent, or explanation for lack thereof; other confidentiality and data security issues	3
**Data collection methods** - Types of data collected; details of data collection procedures including (as appropriate) start and stop dates of data collection and analysis, iterative process, triangulation of sources/methods, and modification of procedures in response to evolving study findings; rationale**	3

For peer review only - http://bmjopen.bmj.com/site/about/guidelines.xhtml

**Data collection instruments and technologies** - Description of instruments (e.g., interview guides, questionnaires) and devices (e.g., audio recorders) used for data collection; if/how the instrument(s) changed over the course of the study	3
**Units of study** - Number and relevant characteristics of participants, documents, or events included in the study; level of participation (could be reported in results)	3,4
**Data processing** - Methods for processing data prior to and during analysis, including transcription, data entry, data management and security, verification of data integrity, data coding, and anonymization/de-identification of excerpts	3
**Data analysis** - Process by which inferences, themes, etc., were identified and developed, including the researchers involved in data analysis; usually references a specific paradigm or approach; rationale**	3
**Techniques to enhance trustworthiness** - Techniques to enhance trustworthiness and credibility of data analysis (e.g., member checking, audit trail, triangulation); rationale**	3

Results/findings

**Synthesis and interpretation** - Main findings (e.g., interpretations, inferences, and themes); might include development of a theory or model, or integration with prior research or theory	4-9
**Links to empirical data** - Evidence (e.g., quotes, field notes, text excerpts, photographs) to substantiate analytic findings	4-9

Discussion

**Integration with prior work, implications, transferability, and contribution(s) to the field -** Short summary of main findings; explanation of how findings and conclusions connect to, support, elaborate on, or challenge conclusions of earlier scholarship; discussion of scope of application/generalizability; identification of unique contribution(s) to scholarship in a discipline or field	9-10
**Limitations** - Trustworthiness and limitations of findings	11

Other

**Conflicts of interest** - Potential sources of influence or perceived influence on study conduct and conclusions; how these were managed	11
**Funding** - Sources of funding and other support; role of funders in data collection, interpretation, and reporting	11

*The authors created the SRQR by searching the literature to identify guidelines, reporting standards, and critical appraisal criteria for qualitative research; reviewing the reference lists of retrieved sources; and contacting experts to gain feedback. The SRQR aims to improve the transparency of all aspects of qualitative research by providing clear standards for reporting qualitative research.

For peer review only

For peer review only - http://bmjopen.bmj.com/site/about/guidelines.xhtml

**The rationale should briefly discuss the justification for choosing that theory, approach, method, or technique rather than other options available, the assumptions and limitations implicit in those choices, and how those choices influence study conclusions and transferability. As appropriate, the rationale for several items might be discussed together.

Reference:

O'Brien BC, Harris IB, Beckman TJ, Reed DA, Cook DA. **Standards for reporting qualitative research: a synthesis of recommendations.** *Academic Medicine*, Vol. 89, No. 9 / Sept 2014 DOI: 10.1097/ACM.0000000000000388

For peer review only

For peer review only - http://bmjopen.bmj.com/site/about/guidelines.xhtml
